# SinGAN-CBAM: a multi-scale GAN with attention for few-shot plant disease image generation

**DOI:** 10.3389/fpls.2025.1703529

**Published:** 2025-11-14

**Authors:** Mengyao Wu, Xinrui Wang, Rongbiao Ji, Yadong Li, Zongyuan Lv, Li Chen, Jianping Yang, Canyu Wang

**Affiliations:** 1 College of Big Data, Yunnan Agricultural University, Kunming, Yunnan, China; 2 Qujing Tobacco Company Shizong Branch, QuJing, Yunnan, China

**Keywords:** generative adversarial networks, SinGAN, attention mechanism, crop disease images, YOLOv8

## Abstract

**Introduction:**

To address the limitation in model performance for tea and coffee disease identification caused by scarce and low-quality image samples, this paper proposes a few-shot multi-scale image generation method named SinGAN-CBAM, aiming to enhance the detail fidelity and semantic usability of generated images.

**Methods:**

The research data were collected from Kunming, Baoshan, and Pu’er regions in Yunnan Province, covering seven typical diseases affecting both tea and coffee plants. Based on the SinGAN framework as the baseline, we incorporate the Convolutional Block Attention Module (CBAM), which leverages dual-channel and spatial attention mechanisms to strengthen the model’s ability to capture texture, edges, and spatial distribution features of diseased regions. Additionally, a SinGAN-SE model is constructed for comparative analysis to evaluate the improvement brought by channel-wise attention mechanisms. The generated images are validated through classification using a YOLO v8 model to assess their effectiveness in real-world recognition tasks.

**Results:**

Experimental results demonstrate that SinGAN-CBAM significantly outperforms GAN, Fast-GAN, and the original SinGAN in metrics such as SSIM, PSNR, and Tenengrad, exhibiting superior structural consistency and edge clarity in generating both tea and coffee disease images. Compared with SinGAN-SE, SinGAN-CBAM further improves the naturalness of texture details and lesion distribution, showing particularly notable advantages in generating complex diseases such as rust and leaf miner infestations. Downstream classification results indicate that the YOLOv8 model trained on data generated by SinGAN-CBAM achieves higher precision, recall, and F1-score than those trained with other models, with key category recognition performance approaching or exceeding 0.98.

**Discussion:**

This study validates the effectiveness of dual-dimensional attention mechanisms in enhancing the quality of agricultural few-shot image generation, providing a high-quality data augmentation solution for intelligent disease identification with promising practical applications.

## 1 Introduction

Tea and coffee are important cash crops in Yunnan Province, possessing significant production value and a broad international consumer market. However, the frequent occurrence of diseases severely threatens their yield and quality, causing not only agricultural economic losses but also weakening the competitiveness of these products in the global market. Therefore, achieving efficient and accurate disease identification and control has become a key challenge in ensuring crop safety and advancing the intelligentization of agricultural production.

In recent years, deep learning technologies have demonstrated great potential in crop disease identification, yet their performance heavily relies on large-scale, high-quality annotated datasets (Keith and Javaan, 2022). In practical agricultural scenarios, acquiring sufficient and diverse disease images faces numerous challenges due to complex shooting conditions and the seasonal and regional nature of disease occurrences, resulting in generally scarce and imbalanced training data. To alleviate this issue, Generative Adversarial Networks (GANs), owing to their powerful data generation capabilities, have gradually been applied to agricultural image enhancement and synthesis tasks, emerging as an effective approach to address few-shot learning problems.

Since the introduction of the GAN framework by Goodfellow et al. in 2014, this technology has rapidly advanced (Goodfellow et al., 2014). Through adversarial training between a generator (G) and a discriminator (D), GANs are able to learn the underlying distribution of real data and generate visually realistic new samples. However, early GANs suffered from issues such as unstable training and mode collapse. To address these problems, a series of improved models have emerged: Deep Convolutional GAN (DCGAN), proposed by Radford et al., enhanced image generation quality by incorporating convolutional architectures (Radford et al., 2015); Conditional GAN (CGAN), introduced by Mirza et al., utilized label information to guide the generation process, mitigating mode collapse, but its applicability is limited due to dependence on annotated data (Mirza and Osindero, 2014); Progressive GAN (PGGAN), developed by Karras et al., successfully generated high-resolution images through a coarse-to-fine multi-scale training strategy, though at the cost of high computational resources and long training times (Karras et al., 2017); subsequently, BigGAN and FastGAN achieved breakthroughs in generation quality and efficiency, respectively, offering new insights for complex image synthesis (Brock et al., 2018; Liu et al., 2021).

In the field of single-image generation, the SinGAN model proposed by Shaham et al. requires only a single natural image for training and achieves image generation from low to high resolution through a multi-scale pyramid structure, demonstrating excellent performance in preserving the original image’s structure and texture details, making it particularly suitable for few-shot agricultural image generation (Shaham et al., 2019). In recent years, research into improving SinGAN has advanced steadily: Nikankin et al. explored diffusion models based on single video frames for generating dynamic content (Nikankin et al., 2022); Hambarde et al. proposed an underwater GAN for single-image depth estimation (Hambarde et al., 2021); Ahmadkhani et al. introduced Topo-SinGAN, incorporating a differentiable topological loss to enhance structural consistency in generated images (Ahmadkhani and Shook, 2024); and Songwei et al. proposed Improved-SinGAN, effectively mitigating geographical bias in remote sensing image generation (Songwei et al., 2021).

In agricultural applications, GANs have been gradually utilized for plant disease detection and phenotypic analysis. Amreen et al. employed C-GAN to generate images of tomato diseased leaves, achieving a classification model accuracy of 98.42% (Amreen et al., 2021); Yuwana et al. expanded a tea dataset using GAN and DCGAN, improving detection model performance by 10%–15% (Yuwana et al., 2020); Cap et al. introduced LeafGAN to transform healthy leaves into diseased ones, significantly enhancing the generalization ability of cucumber disease diagnosis systems (Cap et al., 2020); Hassan et al. developed SRGANs to generate artificial images of young plant seedlings, alleviating issues related to insufficient training data (Hassan et al., 2021); Zhu et al. adopted an improved cDCGAN for grading the vitality of orchid seedlings, verifying the usability of generated images in practical tasks (Zhu et al., 2020).

To assess the practical value of generated images, in addition to using objective metrics such as SSIM, PSNR, and MSE, it is common to verify their semantic consistency and usability through downstream tasks (e.g., classification or detection). The YOLO series of algorithms are widely used in agricultural object recognition due to their efficient and real-time detection capabilities. Among them, YOLOv8 stands out in disease detection tasks with its excellent balance of speed and accuracy, making it suitable as a validation tool for the quality of generated images (Wang et al., 2023).

In summary, although GANs have achieved certain progress in agricultural image generation, research on few-shot disease image generation for specific cash crops such as tea and coffee remains relatively limited. Moreover, existing models still have room for improvement in preserving texture details and representing lesion characteristics. To address these issues, this study focuses on major diseases of tea and coffee, and proposes a single-image generative adversarial network enhanced with attention mechanisms—SinGAN-CBAM. The main research components include: (1) constructing a tea and coffee disease image dataset covering multiple regions in Yunnan Province, and employing instance segmentation techniques to precisely extract lesion areas, thereby eliminating interference from complex backgrounds; (2) comparing the generative performance of GAN, Fast-GAN, and SinGAN under few-shot conditions to establish SinGAN as the baseline model; (3) integrating the CBAM (Convolutional Block Attention Module) and SELayer into SinGAN to enhance feature representation in both spatial and channel dimensions, thereby improving the clarity and realism of generated images; and (4) validating the generated images using YOLOv8 classification to evaluate their effectiveness in downstream recognition tasks. Experimental results demonstrate that SinGAN-CBAM significantly outperforms the original models and SinGAN-SE in both image quality and classification performance, providing an efficient and feasible technical solution for few-shot agricultural image generation, with significant implications for advancing intelligent plant protection and precision agriculture.

## 2 Materials and methods

### 2.1 Experimental materials and data collection

This study focuses on tea and coffee leaves, constructing an image dataset that includes both healthy and typical disease states for the training and evaluation of few-shot disease image generation models.

**Figure 1:**
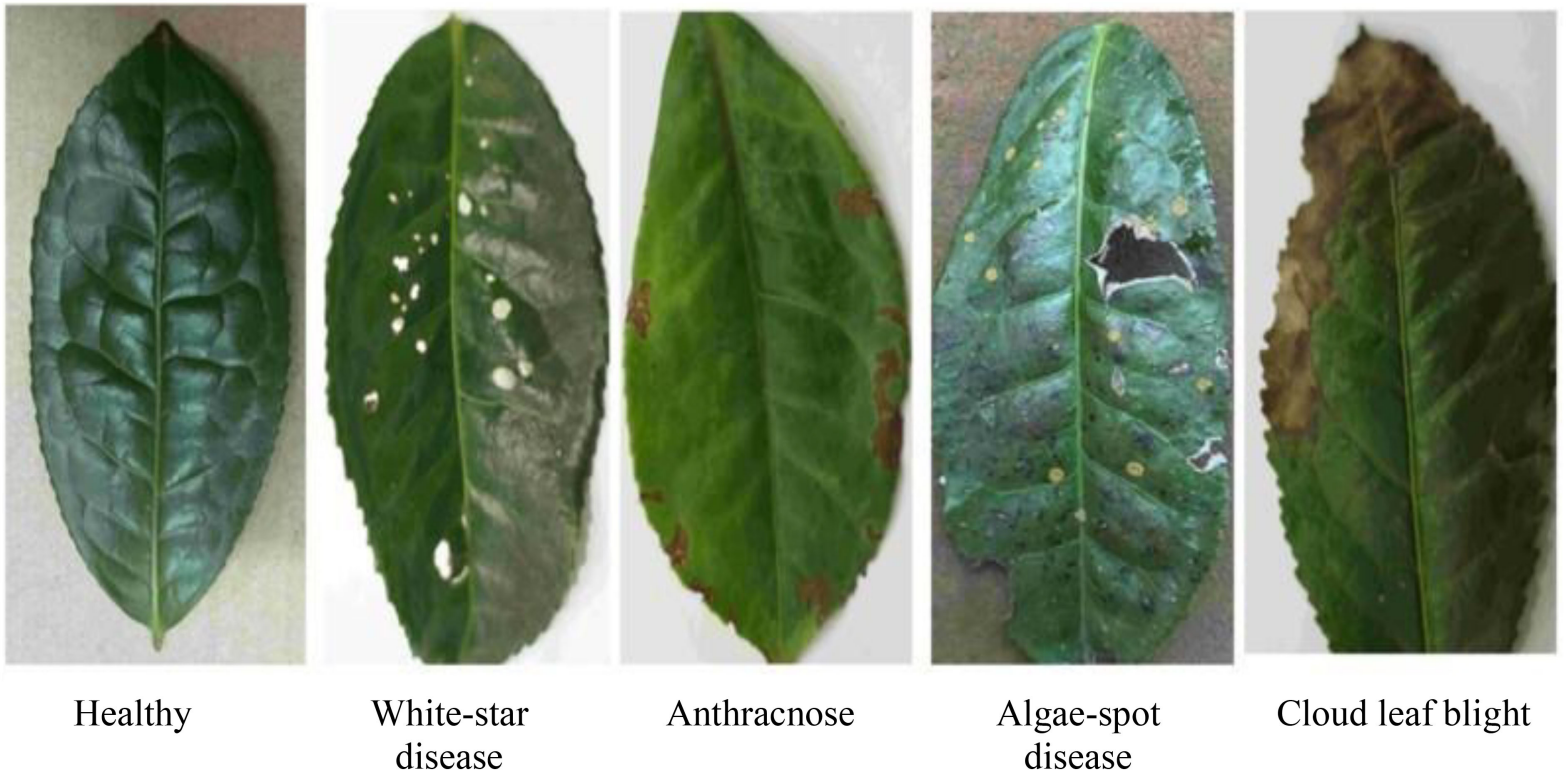
Dataset of different types of tea.

Tea leaf images were collected from the tea plantation located at the back hill of Yunnan Agricultural University in Panlong District, Kunming City, Yunnan Province. Four common diseases that significantly impact tea production were selected as the subjects of this study: White-star disease, Anthracnose, Algae-spot disease, and Cloud leaf blight. Disease images are shown in **Figure 1**. All leaf samples were photographed in the field against a uniform white background using a digital SLR camera, with an image resolution of 5472 × 3648 pixels, ensuring even lighting and no shadow interference. A total of 1075 valid images were collected, categorized to form the tea disease dataset (see **Table 1**).

**Table 1:** Overview of the tea dataset.

Tea types	Healthy	White-star disease	Anthracnose	Algae-spot disease	Cloud leaf blight
Original-quantity	170	188	269	146	302
Filter quantity	100	100	100	100	100

**Figure 2:**
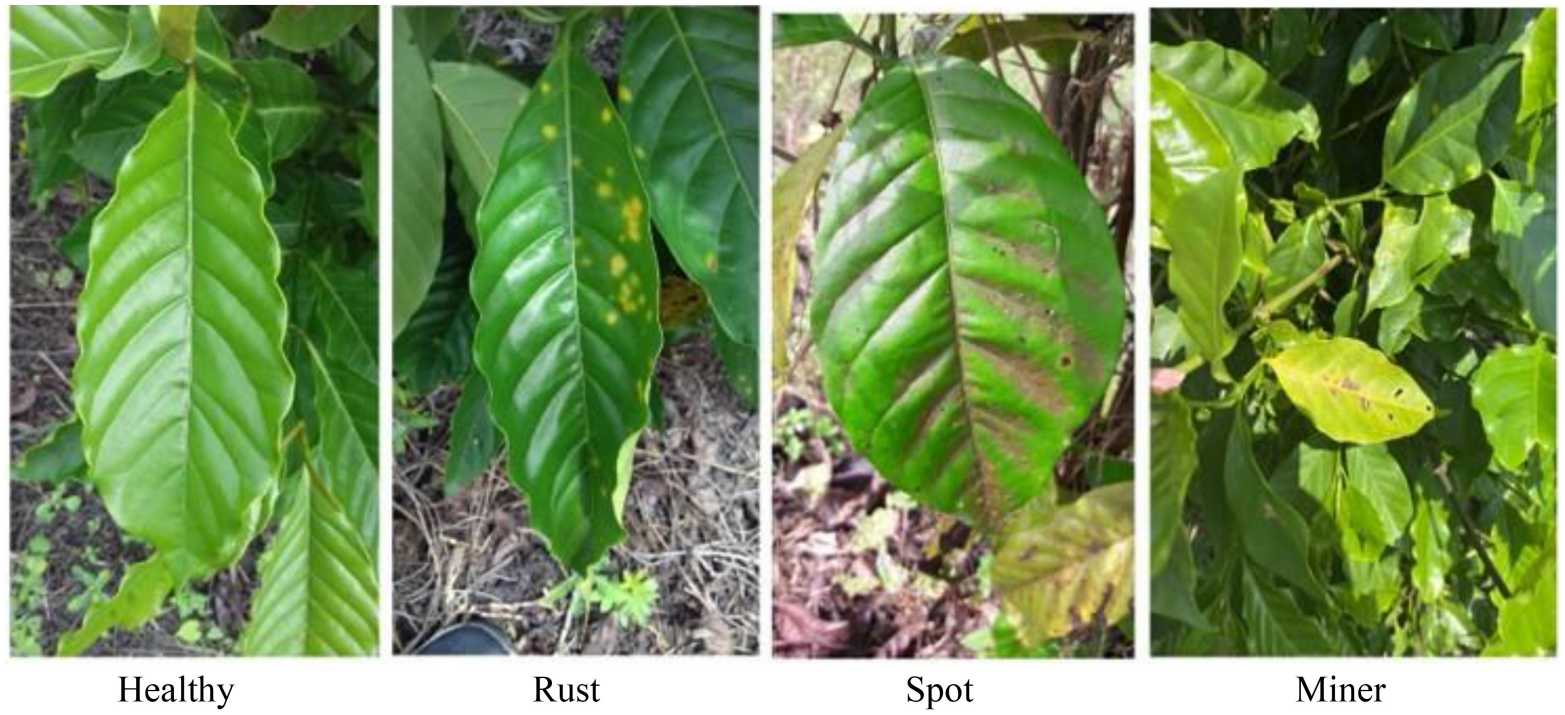
Dataset of different types of coffee.

Coffee leaf images were sourced from two major growing areas: the Lujiang Town Coffee Plantation Base in Baoshan City, Yunnan Province, and the NanDaohe Baisha Slope Coffee Demonstration Base of Yunnan Agricultural University in Pu’er City. The study includes three main diseases: Coffee Rust, Coffee Spot Disease, and symptoms of damage by the Coffee Leaf Miner, with disease images shown in **Figure 2**. Images were taken directly in the field using smartphones under natural lighting conditions, focusing on capturing the leaves of plants in the field, resulting in a total of 749 valid images. During shooting, efforts were made to keep the lens perpendicular to the leaf surface and focus on clear diseased areas to minimize blurriness and obstruction. The final constructed coffee disease dataset is shown in **Table 2**.

**Table 2:** Overview of the coffee leaves dataset.

Coffee types	Healthy	Rust	Spot	Miner
Original quantity	202	247	159	141
Filter quantity	100	100	100	100

All original images were stored in JPEG format and underwent preliminary screening to remove blurry images, those with severe occlusions, or those where non-target areas occupied an excessive proportion, ensuring data quality.

### 2.2 Image preprocessing and instance segmentation

Since the coffee images were taken on-site with complex backgrounds (including branches, soil, adjacent leaves, etc.), this could cause noise interference for the feature extraction of subsequent image generation models. To enhance the semantic consistency of the generated images and the integrity of lesion structures, this study introduces instance segmentation technology to accurately extract target leaves from coffee images.

**Figure 3:**
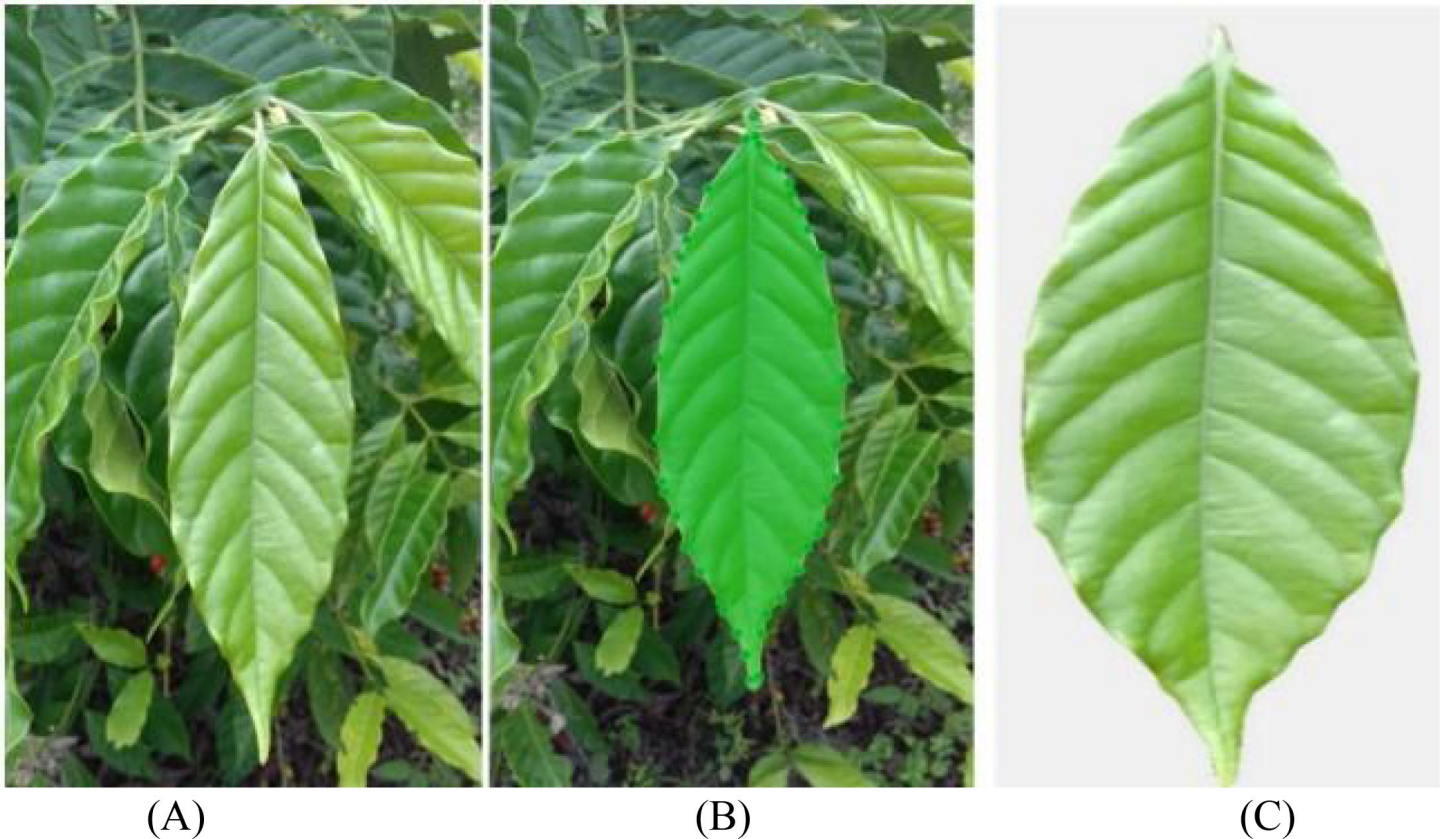
The annotation process and the extracted image. **(A)** Original image; **(B)** Segmentation label; **(C)** Extracted image.

**Figure 4:**
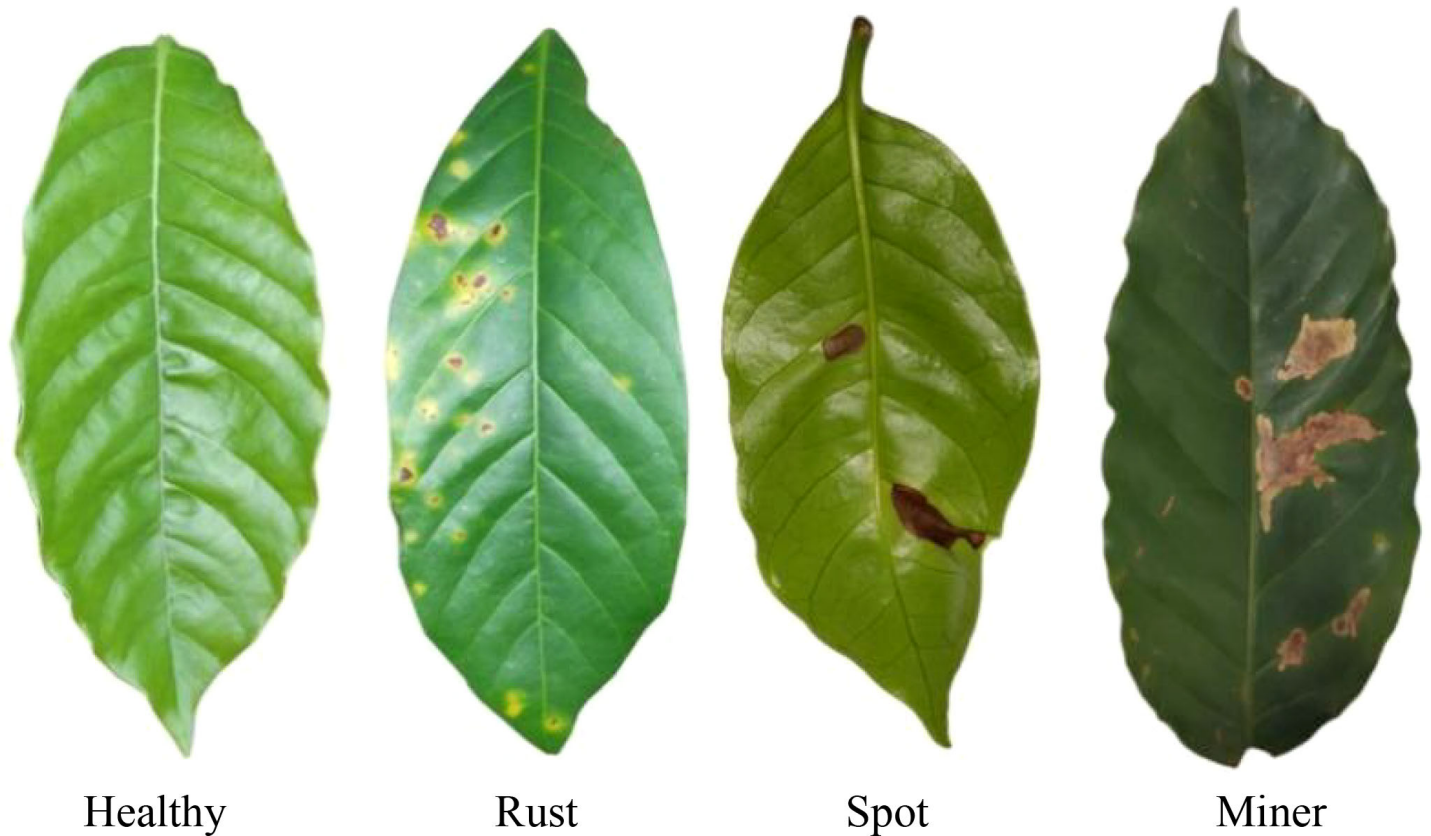
Processed coffee dataset.

The Segment Anything 2.1 (Base) model in the X-AnyLabeling-CPU software platform is utilized for automatic initial segmentation of each coffee image, followed by meticulous annotation completed with manual corrections. For each type of disease image, the prompt point function of the Segment Anything model is used to generate high-quality masks, outputting JSON-formatted annotation files which are stored locally. Subsequently, custom Python scripts are employed to parse the original images and their corresponding JSON files, cropping out areas containing only the target leaves, and standardizing the background to white. This achieves background standardization. **Figure 3** illustrates examples of the segmentation annotation process and extraction results. After processing, a sub-dataset of coffee disease images with unified backgrounds and clear lesions was constructed, with example images for each disease type shown in **Figure 4**. This preprocessing workflow effectively eliminated the interference of complex backgrounds, enhanced the structural consistency of the images, and provided high-quality inputs for the training of subsequent small-sample generation models.

### 2.3 Selection of benchmark generation models

In agricultural image analysis tasks, the acquisition of disease samples is often limited by seasonality, geographic constraints, and the cost of manual labeling, resulting in a limited scale of training data that barely meets the demands of deep generative models for large datasets. To address this challenge, various generative adversarial networks designed for few-sample conditions have been proposed in recent years, showing promising potential in image enhancement and data augmentation. To systematically compare the adaptability and performance of different generation paradigms in the task of generating tea and coffee disease images, this study selects three representative generative models as benchmarks: the standard Generative Adversarial Network (GAN), the lightweight high-resolution generation model (Fast-GAN), and the multi-scale generative network based on single-image (SinGAN).

The standard Generative Adversarial Network (GAN), proposed by Goodfellow et al., achieves mapping from latent space to real data distribution through an adversarial learning mechanism between the generator and discriminator (Goodfellow et al., 2014). This framework laid the foundation for deep generative models and has been widely applied in image synthesis. However, its training process is highly sensitive to sample size, often suffering from issues such as mode collapse and convergence difficulties under few-shot conditions, which limits the diversity of generated images. Although structural improvements (e.g., introducing convolutional architectures) can enhance stability (Radford et al., 2015), its reliance on large-scale training data still restricts direct application in low-resource agricultural scenarios.

Fast-GAN, proposed by Liu et al., is a lightweight framework designed for high-resolution image generation and specifically tailored for few-shot conditions (Liu et al., 2021). The model introduces a Skip-layer Channel Excitation (SLE) module to enhance cross-scale feature representation and employs a self-supervised discriminator to improve discrimination capability, enabling fast convergence and stable training even with limited samples. It has demonstrated superior efficiency and robustness compared to StyleGAN2 in tasks involving material images and natural scene generation, making it suitable for moderate-scale few-shot image generation. Therefore, it is included in the comparative framework of this study.

SinGAN represents an even more extreme few-shot generation paradigm—requiring only a single training image to construct a multi-scale generative network (Shaham et al., 2019). The model builds a pyramid structure, training independent generator-discriminator pairs at multiple resolution levels to achieve coarse-to-fine image synthesis. Each level learns the local texture and structural statistical characteristics of the input image and generates visually diverse new samples by incorporating noise inputs. Due to its independence from class labels or large datasets, and its ability to effectively preserve the semantic structure of the original image, SinGAN demonstrates unique advantages in tasks involving scarce and structurally complex samples, such as agricultural disease images.

### 2.4 Generator model improvement based on channel attention: SinGAN-SE

To enhance the model’s ability to represent key features of tea and coffee diseases, this study introduces a channel attention mechanism into the original SinGAN framework, proposing an improved model named SinGAN-SE. The model embeds a Squeeze-and-Excitation block (SE Block) (Hu et al., 2018) after each multi-scale convolutional module in both the generator and discriminator, enabling adaptive reweighting of feature channels.

**Figure 5:**
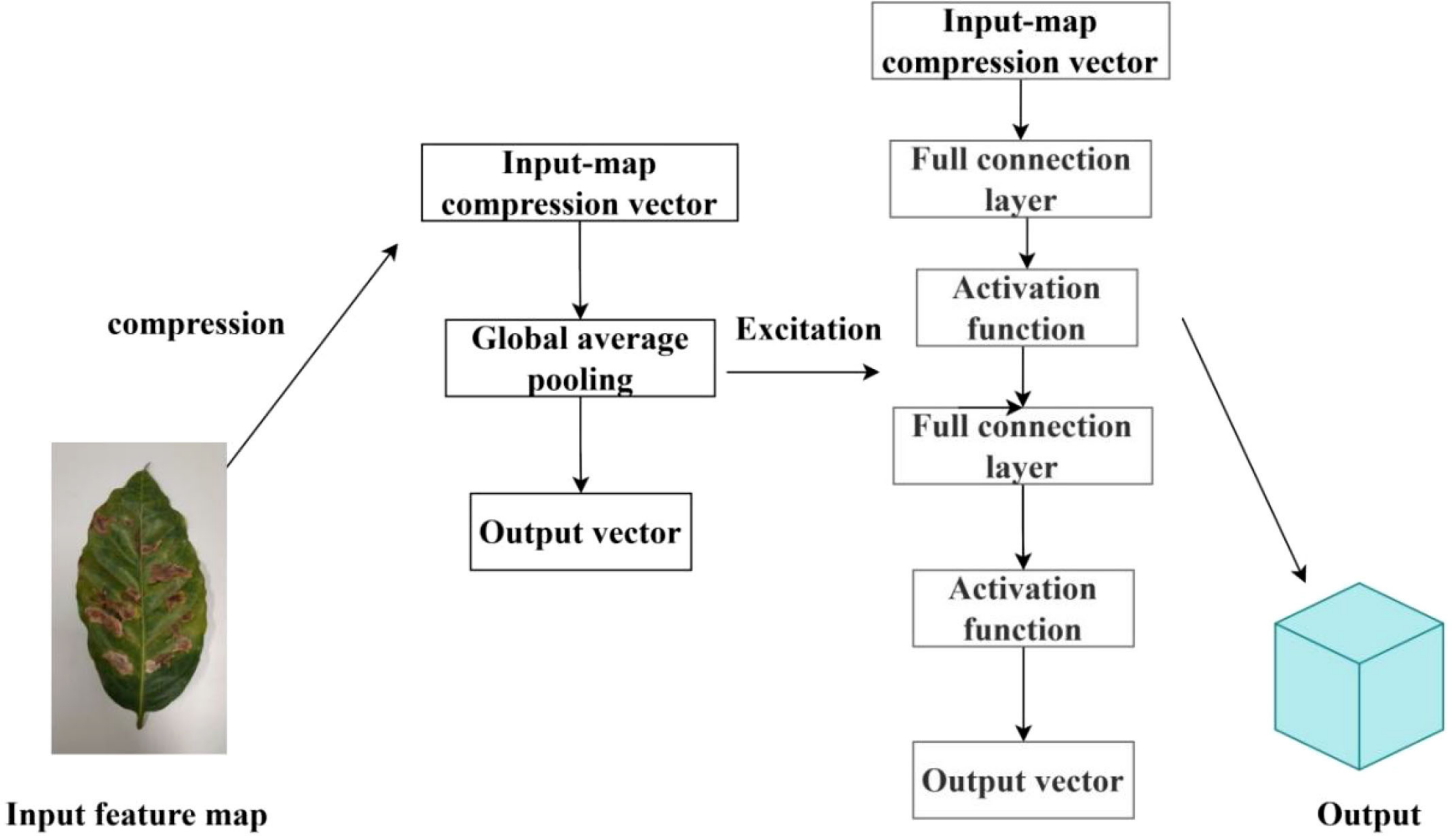
SELayer structure.

**Figure 6:**
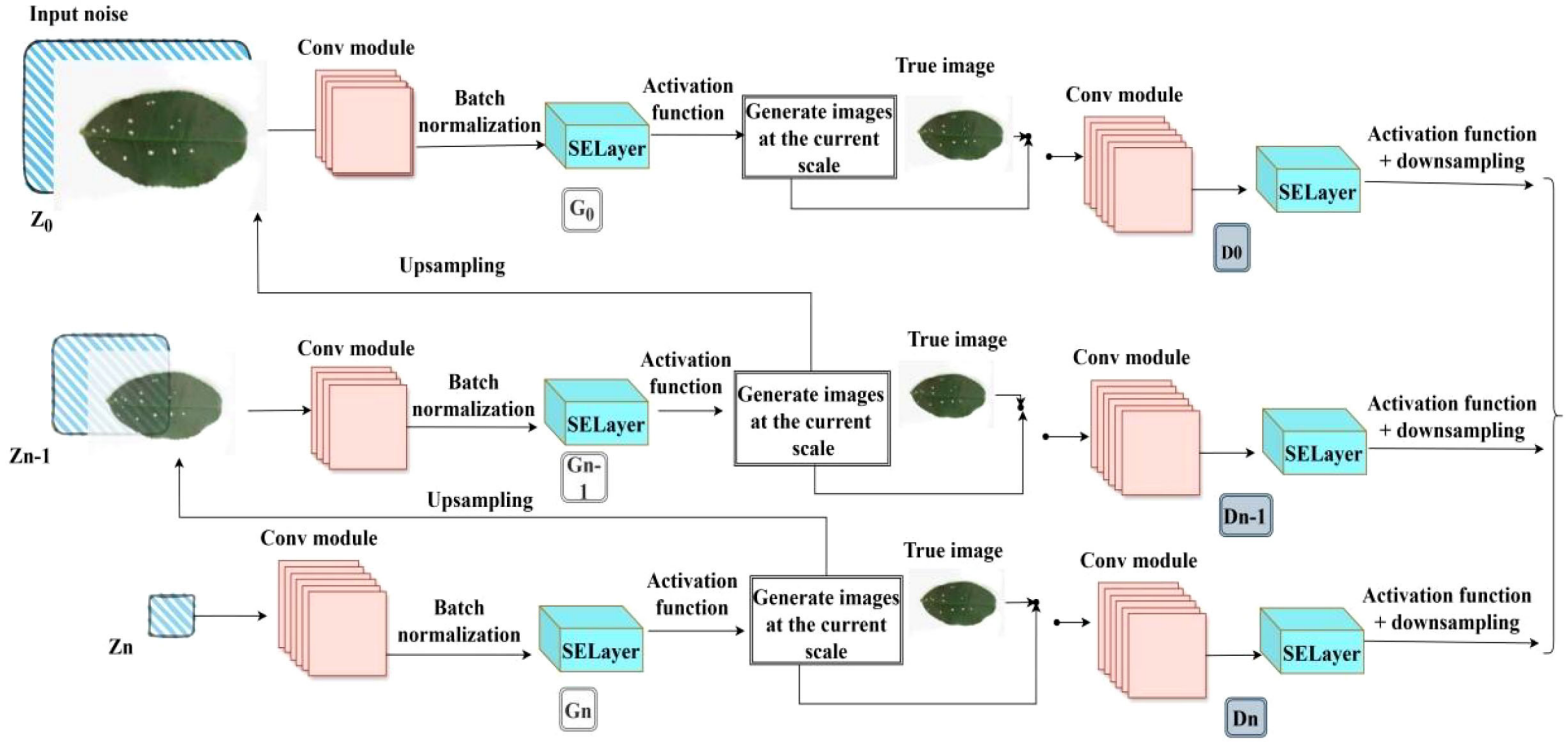
Improved SinGAN-SELayer structure.

The SE block “squeezes” spatial information through a global average pooling operation to generate channel-wise descriptors, then learns inter-channel dependencies via fully connected layers, and finally outputs a set of weighting coefficients to “re-calibrate” the feature maps across channels. This mechanism enables the model to dynamically enhance responses from channels that are important for disease texture representation, while suppressing interference from background or irrelevant textures, thereby improving the discriminative power of feature representation under few-shot conditions. The architecture of the SELayer is shown in **Figure 5**, and the overall structure of the proposed SinGAN-SE model is illustrated in **Figure 6**.

In SinGAN-SE, the SE module is integrated after every residual block in generators and discriminators at each scale, forming a “convolution-attention” cascading structure. The training process follows SinGAN’s progressive growing strategy: starting from the lowest resolution and gradually moving upwards, with parameters being fixed once the training of each layer is completed. The loss function at each scale consists of an adversarial loss and a reconstruction loss: the adversarial loss maintains the balance between the generator and discriminator; the reconstruction loss uses the L1 norm to constrain the consistency between the generated image and the input image in the pixel space, ensuring that the generation result retains the original structure.

Parameter updates use an alternating optimization strategy: in each iteration round, the discriminator is optimized first, followed by the generator, to maintain training stability. This structure enhances the model’s selective response capability to disease-related channel features without significantly increasing the computational burden.

### 2.5 Generator model improvement based on dual-dimensional attention: SinGAN-CBAM

To further enhance the model’s spatial localization and structural perception capabilities for diseased regions, this study explores a fusion strategy incorporating both channel and spatial attention mechanisms, proposing the SinGAN-CBAM model. The model introduces the Convolutional Block Attention Module (CBAM) (Woo et al., 2018) into the generator and discriminator at each scale of SinGAN. By cascading channel and spatial attention sub-modules, CBAM enables joint optimization of feature maps.

**Figure 7:**
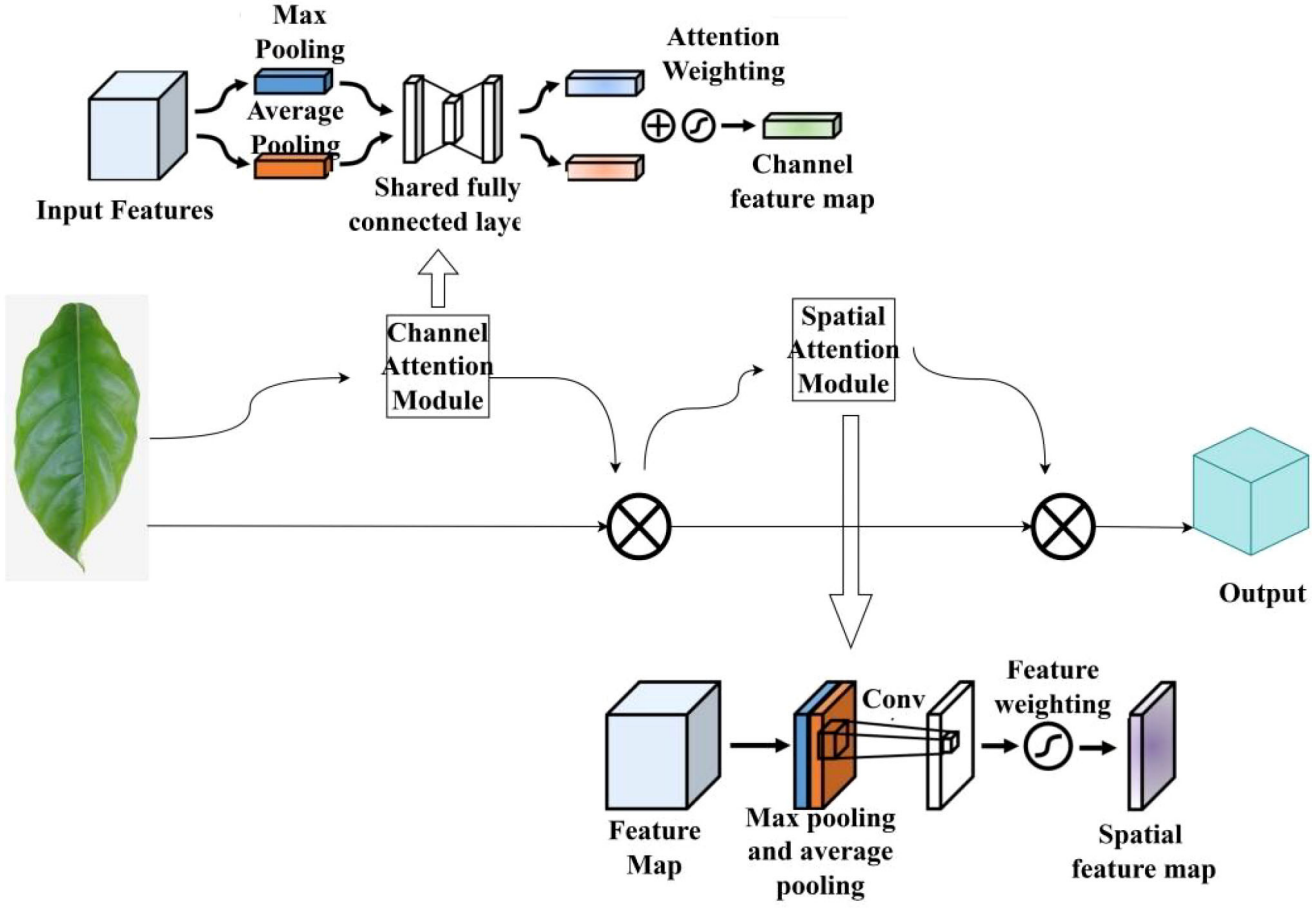
Structure of CBAM.

CBAM first computes the importance weights of each feature channel through the channel attention sub-module. This mechanism is similar to SE, but employs dual pathways of max pooling and average pooling to enhance feature representation. Subsequently, the spatial attention sub-module calculates spatial position weights based on the channel-aggregated feature map, amplifying the response strength of key regions, thereby more precisely capturing the spatial distribution patterns of diseases. The architecture of CBAM is illustrated in **Figure 7**.

**Figure 8:**
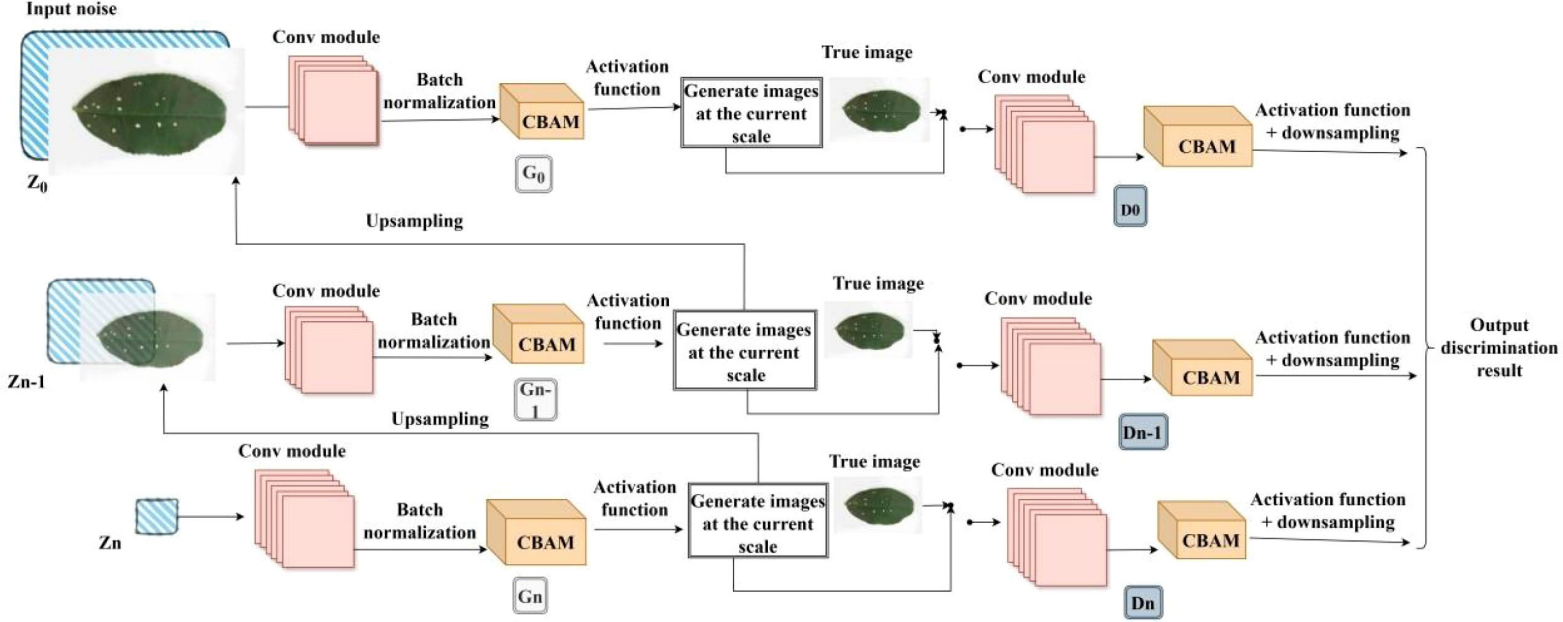
Improved SinGAN-CBAM structure.

In SinGAN-CBAM, the CBAM module is embedded after each convolutional block in both the generator and discriminator, participating in the entire generation process from low to high resolution. This design helps mitigate the loss of fine-grained textures in multi-scale generation, particularly enhancing the modeling capability of local details such as lesion edges and color transitions at high-resolution stages. The training strategy remains consistent with that of SinGAN-SE, employing a layer-by-layer training and alternating optimization approach to ensure training stability even after the integration of attention mechanisms. The architecture of the improved model is illustrated in **Figure 8**.

Compared to SinGAN-SE, which focuses only on the channel dimension, SinGAN-CBAM is expected to generate images with higher semantic consistency under complex backgrounds and irregular lesion shapes by leveraging synergistic dual-dimensional attention, thereby providing higher-quality data support for subsequent recognition tasks.

### 2.6 Disease classification performance validation model: YOLOv8

To evaluate the effectiveness of generated images in practical recognition tasks, this study employs YOLOv8 as the downstream disease classification model to verify the performance improvement brought by the generated data. As an efficient object detection framework, YOLOv8 features a backbone network based on CSPDarknet53 and incorporates the Spatial Pyramid Pooling with Fast (SPPF) module, endowing it with strong multi-scale feature extraction capabilities. Compared to traditional classification networks and two-stage detectors, YOLOv8 adopts a single-stage, end-to-end architecture that unifies object localization and category recognition into a single model, significantly improving inference efficiency and making it well-suited for real-time disease identification in agricultural scenarios.

In this experiment, YOLOv8 is used to perform multi-class disease classification tasks on tea and coffee leaves. The model input consists of a training set composed of a mixture of original and generated images, and the output is the classification results for healthy leaves and various disease categories. By comparing the precision, recall, and F1-score of YOLOv8 when trained on datasets augmented with images generated by different models (GAN, Fast-GAN, SinGAN, SinGAN-SE, SinGAN-CBAM), the practical value of each generation method in enhancing recognition performance can be objectively evaluated.

This validation process not only evaluates the visual quality of the generated images, but also focuses on their semantic usability—that is, whether the generated samples contain discriminative features that can be effectively utilized by recognition models—thereby providing a practical evaluation criterion for the agricultural application of generative models.

### 2.7 Evaluation metrics

#### 2.7.1 SSIM

SSIM is designed based on three factors: brightness, contrast, and structure to better adapt to the working principle of the human visual system. SSIM measures the similarity between two images. The SSIM value is specified in [0,1], and the size of the value represents the effect of the image. The larger the value, the better the image effect, and the smaller the value, the worse the image effect. The mathematical calculation formula of SSIM is (Equation 1):

(1) $$\begin{array}{c} SSIM(x,y)={\left[C_{\iota }(x,y)\right]}^{\alpha }{\left[C_{c}(x,y)\right]}^{\beta }{\left[C_{s}(x,y)\right]}^{\gamma } \end{array}$$

where x,y represent the real image and the generated image, and α、 β、 γ represent the controllable parameters. When α= β= γ=1, we can get (Equation 2):

(2) $$\begin{array}{c} SSIM(x,y)=\frac{\left(2\mu_{x}\mu_{y}+C_{1})(2\sigma_{xy}+C_{2}\right)}{\left(\mu_{x}^{2}+\mu_{y}^{2}+C_{1})(\sigma_{x}^{2}+\sigma_{y}^{2}+C2\right)} \end{array}$$

#### 2.7.2 MSE

MSE is the most commonly used estimator in image quality measurement. It is a complete reference indicator that calculates the average value of the difference in pixel values ​​between two images. The closer the value is to zero, the better. MSE introduces the root mean square error (RMSE) or root mean square deviation (RMSD), which is often referred to as the standard deviation of the variance (Equation 3).

(3) $$\text{MSE}=\frac{1}{MN}\sum_{i=1}^{M}\sum_{j=1}^{N}{\left[I_{1}\left(i,j\right)-I_{2}\left(i,j\right)\right]}^{2}$$

Where ${\text{I}}_{1}$ and ${\text{I}}_{2}$ are two images, M and N are the height and width of the images respectively, and i and j are the pixel position indexes.

#### 2.7.3 PSNR

PSNR is used to calculate the ratio between the maximum possible signal power and the distortion noise power that affects its representation quality. Peak signal-to-noise ratio is the most commonly used quality assessment technique to measure the reconstruction quality of lossy image compression codecs. The signal is regarded as raw data, and the noise is the error caused by compression or distortion. The image quality is evaluated by measuring the error between the reconstructed image and the reference image. It is based on MSE, which is the square average of the difference between each pixel. The larger the PSNR value, the smaller the difference between the generated image and the input real image, and the better the image quality. The formula is as follows (Equation 4):

(4) $$\begin{array}{c} PSNR=10\cdot log_{10}(\frac{MAX^{2}}{MSE}) \end{array}$$

where MAX is the maximum possible pixel value of the image (e.g., for an 8-bit image, MAX = 255), and MSE is the mean squared error between the reconstructed image and the reference image.

#### 2.7.4 Tenengrad

The Tenengrad gradient method uses the Sobel operator to calculate the gradients in the horizontal and vertical directions respectively. In the same scene, the higher the gradient value, the clearer the image. The calculation is the sum of the squares of the gradients of the image after being processed by the Sobel operator or other edge detection operators. The larger the gradient value, the clearer the image texture.

The gradient calculation formula of image I at point ( x,y) is as follows, where ${\text{G}}_{\text{x}}$ and ${\text{G}}_{\text{y}}$ are Sobel’s convolution kernels (Equation 5).

(5) $$\begin{array}{c} S_{\left(x,y\right)}=\sqrt{G_{x}*I(x,y)+G_{y}*I(x,y)} \end{array}$$

The Tenengrad value of this image is (Equation 6):

(6) $$Ten=\frac{1}{n}*\sum_{x}\sum_{y}S{\left(x,y\right)}^{2}$$

In the disease classification task of YOLOv8, the classification results are comprehensively evaluated through multi-dimensional indicators. The key evaluation indicators used are Precision, Recall, and F1-score measures the proportion of correctly predicted samples to the total samples; precision, also known as the search rate, is the proportion of all samples predicted to be positive that are actually positive; recall, also known as the search rate, is the proportion of all samples that are actually positive that are correctly identified as positive. The three formulas are as follows (Equations 7–9):

(7) $$\begin{array}{c} Precision=\frac{TP}{TP+FP} \end{array}$$

(8) $$\begin{array}{c} Recall=\frac{TP}{TP+FN} \end{array}$$

(9) $$\begin{array}{c} F1-score=2\cdot \frac{Precision\cdot Recall}{Precision+Recall} \end{array}$$

## 3 Experiment and result analysis

### 3.1 Experimental environment and parameter configuration

The experiment was conducted in a MobaXterm terminal environment, on a hardware platform equipped with an AMD Ryzen 5 3500U processor and a single NVIDIA GeForce RTX 4090 graphics card, with CUDA version 11.5. The software environment was built based on Python 3.9.18, and the deep learning framework used was PyTorch 2.0.0. Detailed environment configuration and hyperparameter settings are shown in **Table 3**, respectively.

**Table 3:** Parameter settings.

Parameter	GAN	Fast-GAN	SinGAN
Learning Rate	0.002	0.002	0.002
Optimizer	Adam	Adam	Adam
Batch-Size	4	4	1
Iteration steps	2000	2000	2000

### 3.2 Comparison of generation effects of benchmark models

**Figure 9:**
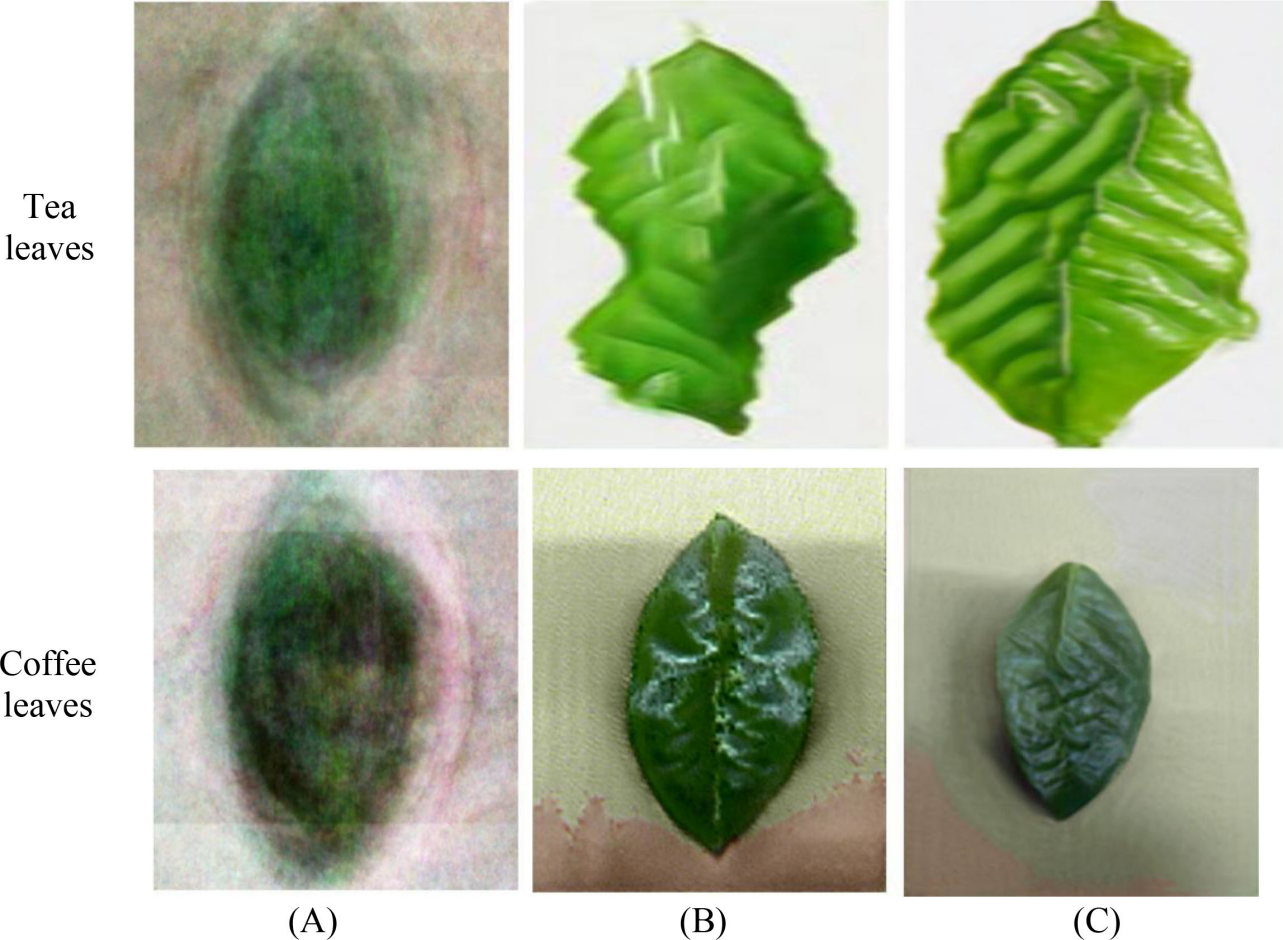
Example of the generated image of healthy tea and coffee leaves. **(A) **GAN-generated images; **(B) **Fast-GAN-generated images; **(C) **SinGAN-generated images.

To evaluate the performance of different generation models on the task of generating images of tea and coffee plant diseases, this paper trains and generates images using three benchmark models: GAN, Fast-GAN, and SinGAN. **Figure 9** shows the generation results of healthy leaves of tea and coffee.

From a visual perspective, GAN-generated images exhibit obvious defects: blurred edges, coarse details, failure to clearly render the serrated leaf margins, and lack of the sharp texture characteristic of real leaves, resulting in significant overall differences from real images. This is primarily due to GAN’s weak ability to capture high-frequency texture information during training, making it difficult to effectively model edge and microstructure features. Fast-GAN shows improvement in edge clarity, with textures emerging in some areas, but still suffers from artifacts and unnatural texture distribution, indicating limited accuracy in reconstructing local details.

In contrast, SinGAN leverages its multi-scale pyramid structure to progressively capture both global layout and local texture features, achieving significantly better detail representation than the other two models. Leaf color reproduction is accurate, vein textures are clear, surface highlights and shadow transitions appear natural, and the overall visual consistency is higher, demonstrating superior structural fidelity.

Although visual evaluation provides an intuitive reference, subjective judgment is susceptible to individual bias. Therefore, to further objectively assess generation quality, this study employs Structural Similarity (SSIM), Mean Squared Error (MSE), Peak Signal-to-Noise Ratio (PSNR), and Tenengrad gradient values as quantitative evaluation metrics. The average metric values for each model are presented in **Table 4**.

**Table 4:** Evaluation index results of images generated by different models.

Evaluation index	Study subjects	GAN	Fast-GAN	SinGAN
SSIM	Tea	0.284	0.552	**0.682**
	Coffee	0.279	0.530	**0.636**
MSE	Tea	70.35	69.86	**46.97**
	Coffee	79.83	88.95	**48.48**
PSNR	Tea	29.66	29.68	**31.41**
	Coffee	29.11	28.64	**31.27**
Tenengrad	Tea	0.201	0.359	**0.567**
	Coffee	0.197	0.301	**0.454**

The bolded numbers in the table represent the optimal results (values).

Comprehensive metric analysis shows that SinGAN outperforms GAN and Fast-GAN across all evaluation dimensions, demonstrating superior generation performance. In tea image generation, SinGAN achieves a 23.6% improvement in SSIM over Fast-GAN, a 32.8% reduction in MSE, and a PSNR of 31.28, representing a 5.9% increase; for coffee images, SSIM improves by 20.0%, MSE decreases by 45.5%, and PSNR increases by 7.4% to 30.70. Furthermore, SinGAN’s Tenengrad value is significantly higher than that of the other models, indicating sharper edges in the generated images. These results validate SinGAN’s superiority in few-shot agricultural image generation tasks and confirm its better suitability for the target crop data characteristics in this study.

### 3.3 Improved effect of introducing channel attention mechanism (SinGAN-SE)

**Figure 10:**
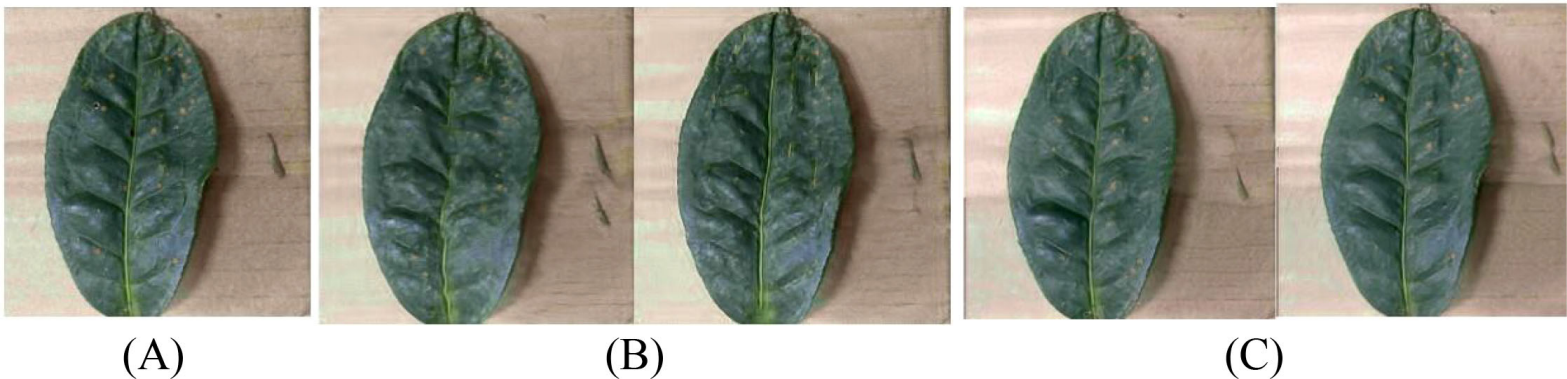
Generated images of white spot disease of tea leaves. **(A) **Original image; **(B) **SinGAN-generated images; **(C) **SinGAN-SELayer-generated images.

**Figure 11:**
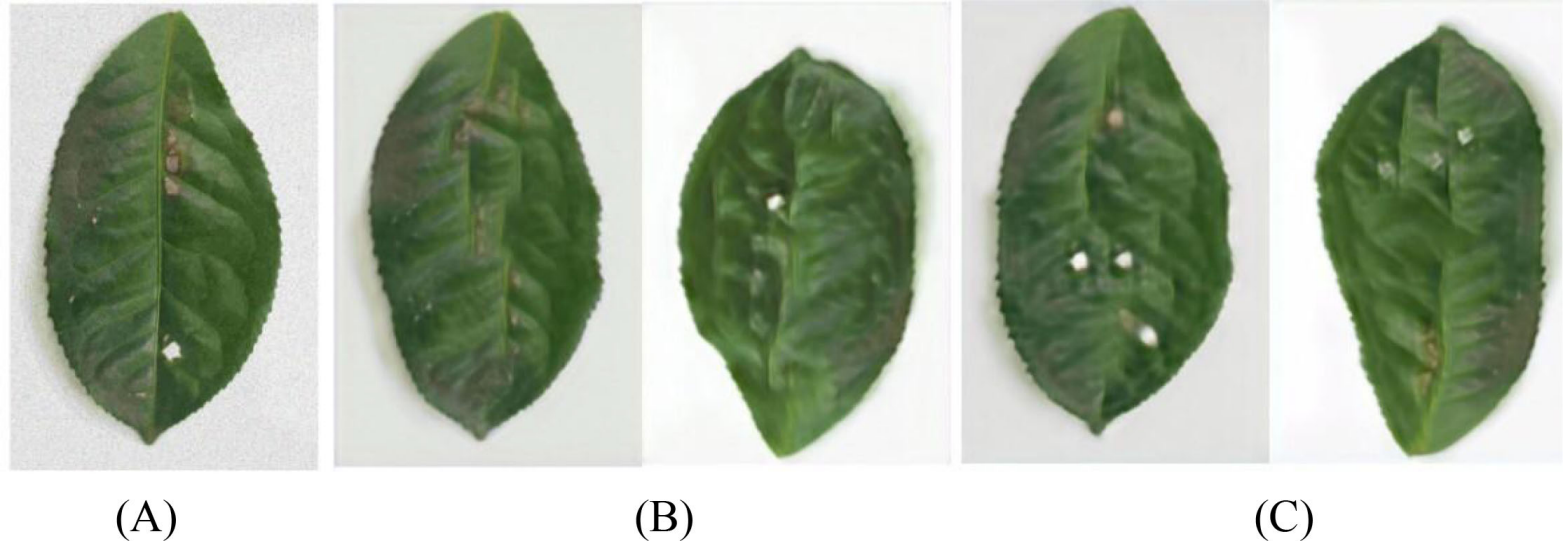
Generated images of algal spot disease of tea leaves. **(A) **Original image; **(B) **SinGAN generated images; **(C) **SinGAN-SE generated images.

In order to further enhance the expression ability of generated images on disease characteristics, this paper introduces the Squeeze-and-Excitation block (SELayer) on the basis of SinGAN to construct an improved model called SinGAN-SE. **Figures 10** and **11** show the generation results of tea white star disease and algal spot disease, respectively.

In the white star disease generation task, the white spots generated by the original SinGAN have blurry edges, insufficient contrast, and a distribution that lacks naturalness. In contrast, the spots generated by SinGAN-SE have sharper boundaries, enhanced color saturation, a density of distribution closer to real diseases, richer textural details, presenting a stronger “graininess” and sense of hierarchy. In the generation of algal spot disease, SinGAN-SE also shows superior shape fidelity and textural coherence.

**Figure 12:**
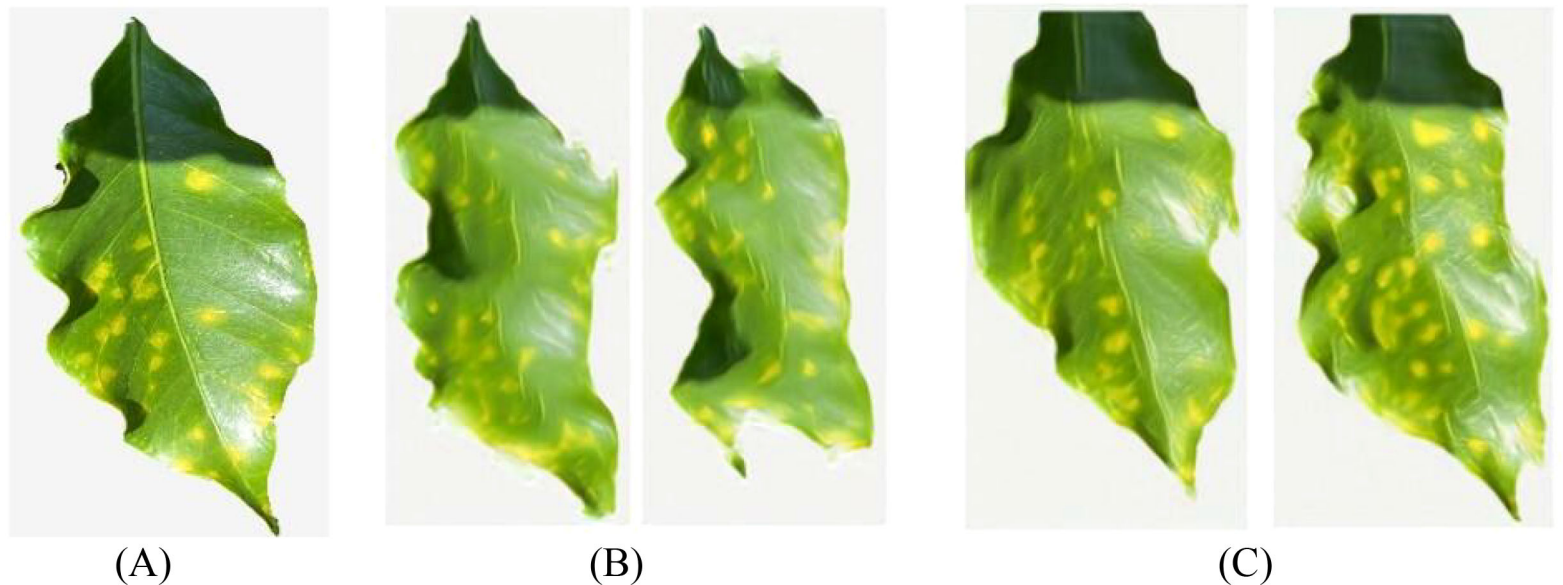
Generated images of coffee leaf rust disease. **(A) **Original image; **(B) **SinGAN generated images; **(C) **SinGAN-SE generated images.

**Figure 13:**
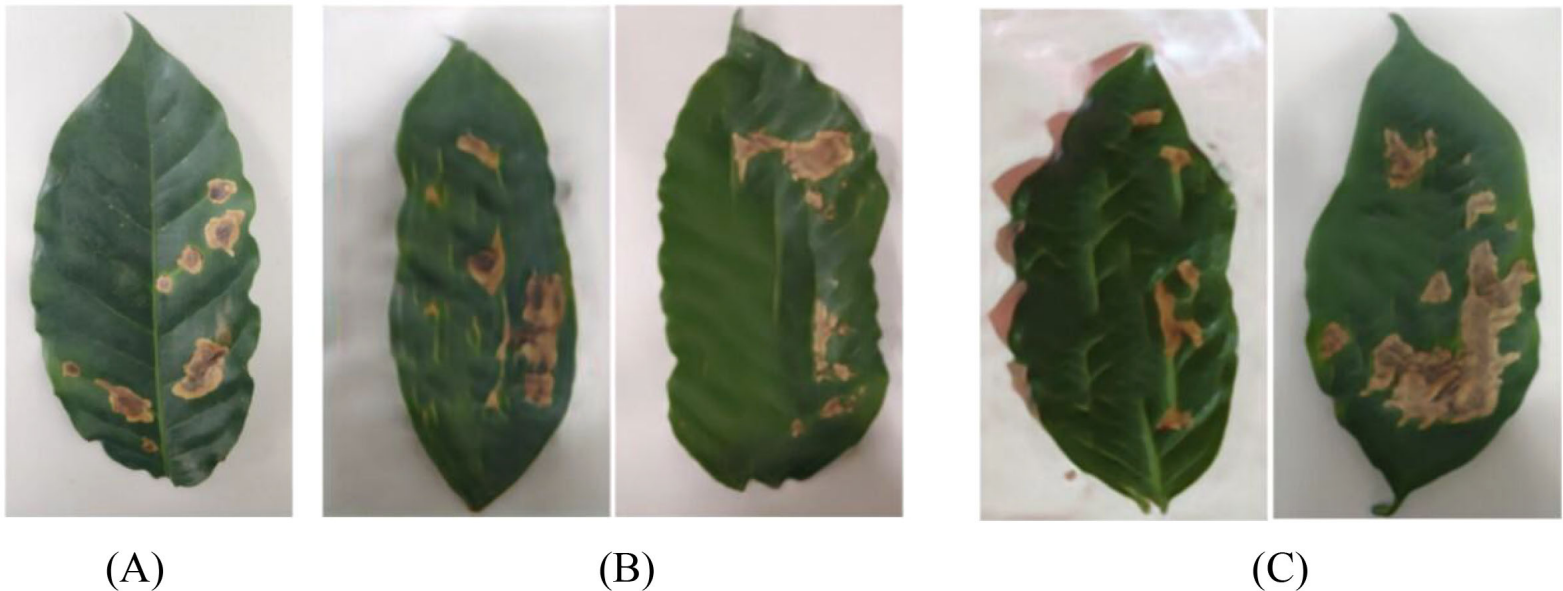
Generated images of the damage caused by the coffee leaf miner. **(A) **Original image; **(B) **SinGAN generated images; **(C) **SinGAN-SE generated images.

For coffee disease images, **Figures 12** and **13** show the generation results of leaf rust and leaf miner damage, respectively. The original SinGAN has issues such as clumped spots and overly dense distribution in generating leaf rust, while the brown patches of leaf miner damage have blurry edges and distorted textures. By reinforcing the response of disease-related features through the channel attention mechanism, SinGAN-SE effectively alleviates these problems: the distribution of rust spots is more natural with smoother edge transitions; the edges of leaf miner patches are clearer, with more realistic color gradients, significantly enhancing overall realism.

To quantify the improvement effects of the SELayer, this paper conducts a quantitative evaluation of the generation results of SinGAN and SinGAN-SE on five types of tea disease images and four types of coffee disease images. The results are shown in **Table 5** and **Table 6**, respectively.

**Table 5:** Quantitative evaluation of the quality of tea generation images.

Evaluation index	SinGAN	SinGAN-SE
SSIM	0.682	0.714
MSE	46.97	32.83
PSNR	31.41	32.97
Tenengrad	0.567	0.664

**Table 6:** Quantitative evaluation of the quality of coffee generation images.

Evaluation index	SinGAN	SinGAN-SE
SSIM	0.636	0.691
MSE	48.48	41.18
PSNR	31.27	31.98
Tenengrad	0.454	0.602

In tea images, the SSIM of SinGAN-SE increases to 0.714 (+4.7%), the MSE decreases by 30.1%, and the PSNR improves to 32.97 (+5.0%). Additionally, the Tenengrad value increases by approximately 17.1%, indicating significant improvements in image structure, clarity, and visual quality. In coffee images, there is an increase in SSIM, a reduction of MSE by about 15%, an improvement of 2.27% in PSNR, and a higher Tenengrad value, suggesting that the generated images surpass the baseline model in pixel-level similarity and detail sharpness. Experimental results demonstrate that the introduction of SELayer effectively enhances the model’s ability to learn key disease features while retaining SinGAN’s multi-scale generation advantages.

### 3.4 Improvement effects of introducing dual-dimensional attention mechanism (SinGAN-CBAM)

**Figure 14:**
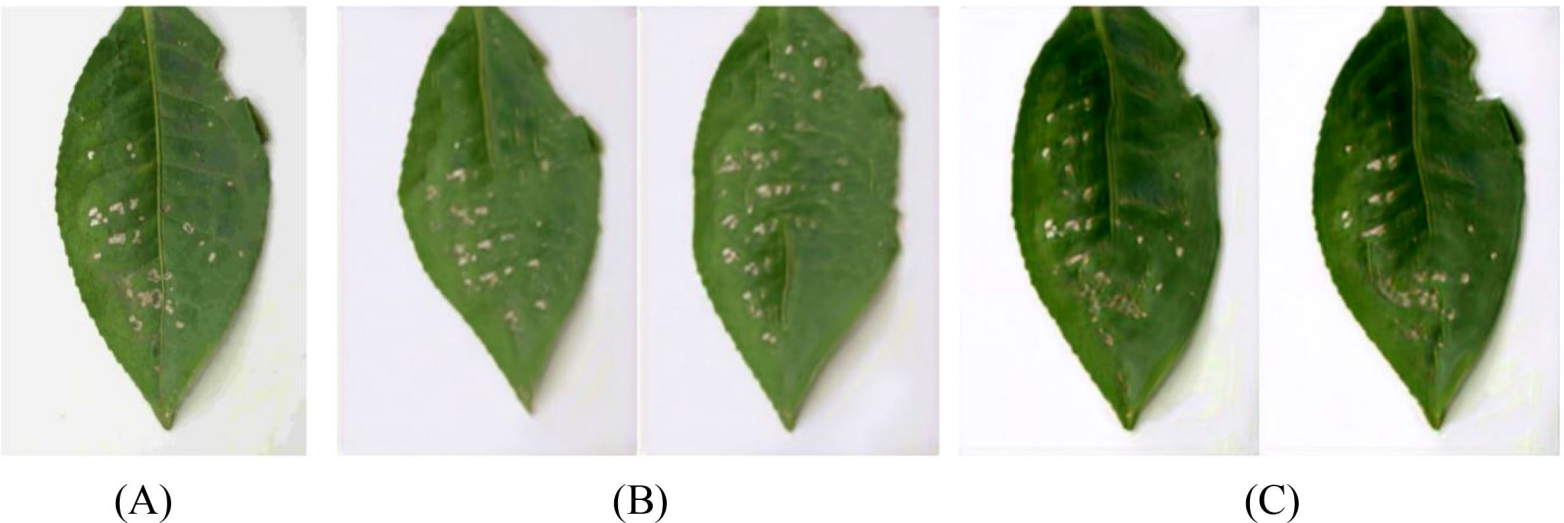
Generated images of white spot disease of tea leaves. **(A)**Original image; **(B)**SinGAN generated images; **(C)**SinGAN-CBAM generated images.

**Figure 15:**
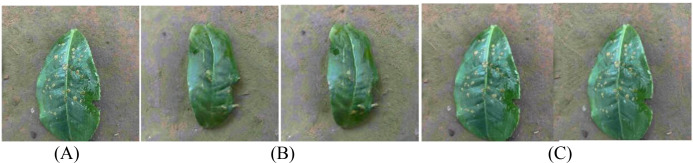
Generated images of algal spot disease of tea leaves. **(A) **Original image; **(B) **SinGAN generated images; **(C) **SinGAN CBAM-generated images.

**Figure 16:**
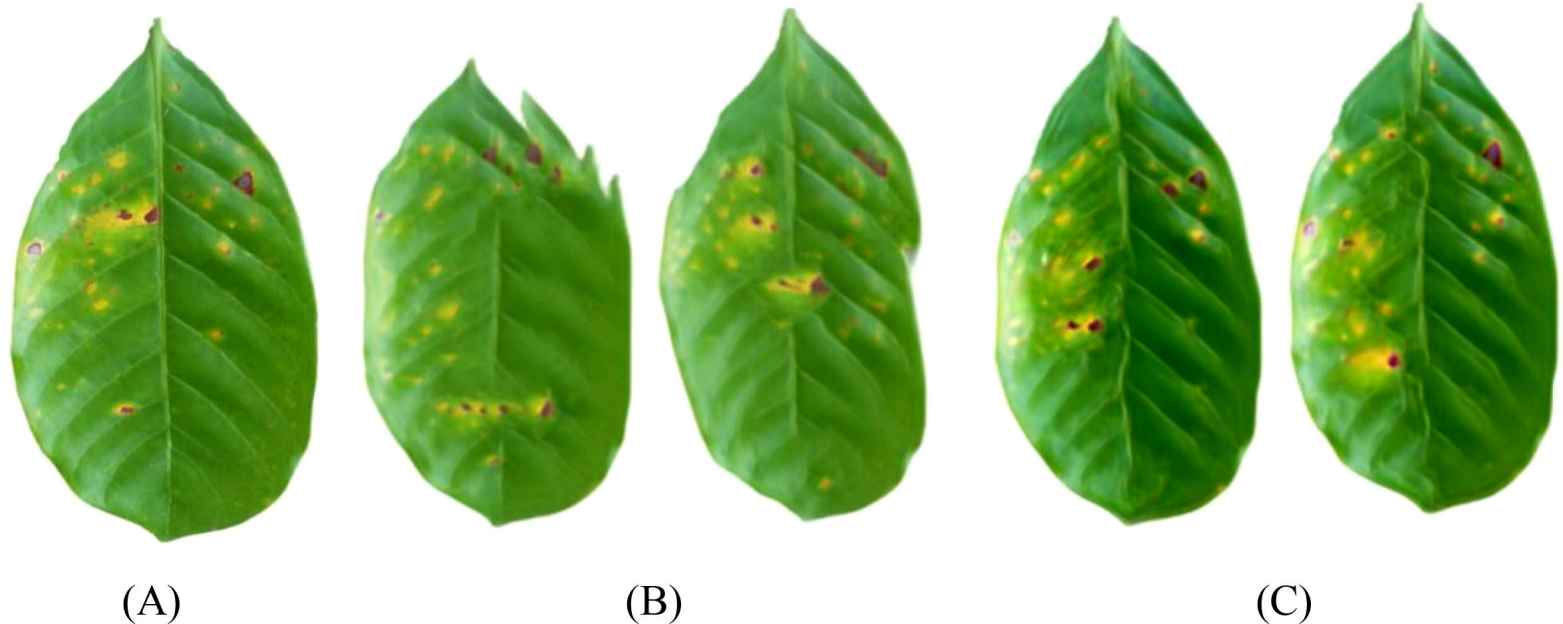
Generated images of coffee leaf rust disease. **(A) **Original image; **(B) **SinGAN generated images; **(C) **SinGAN-CBAM generated images.

**Figure 17:**
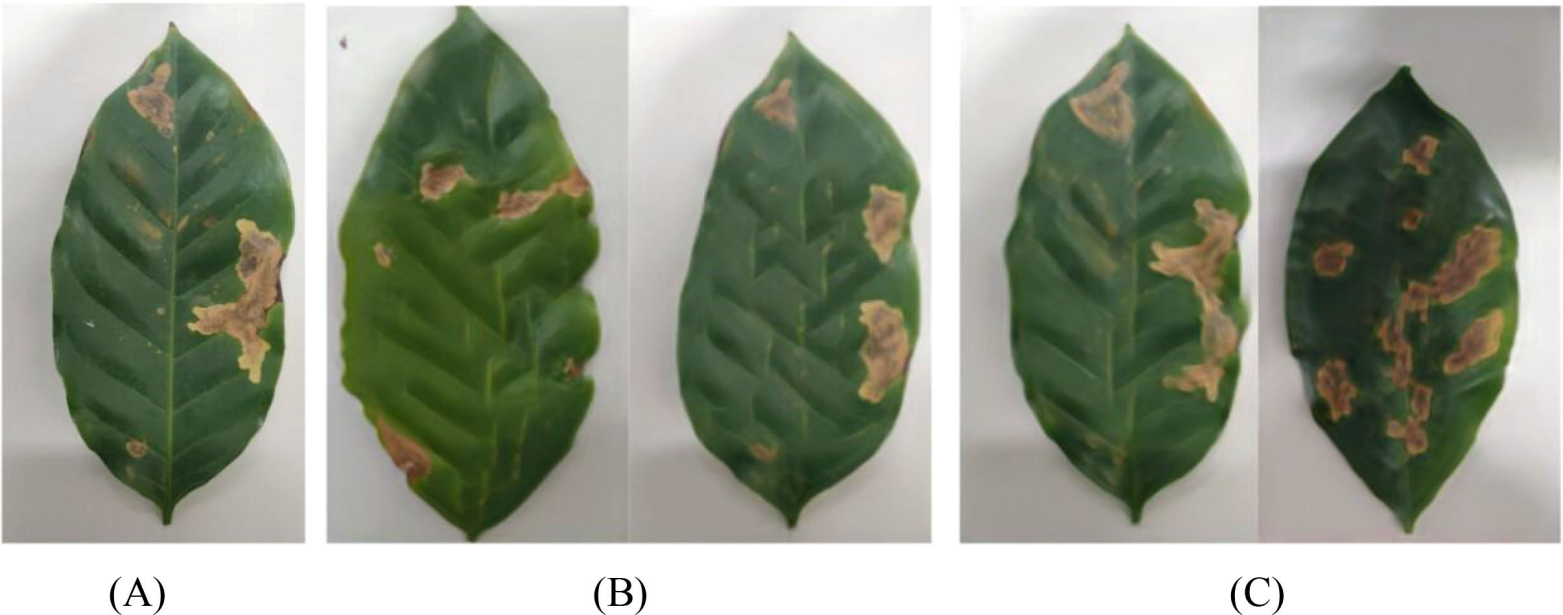
Generated images of the damage caused by the coffee leaf miner. **(A) **Original image; **(B) **SinGAN generated images; **(C) **SinGAN-CBAM generated images.

To further explore the impact of spatial context information on generation quality, this paper constructs the SinGAN-CBAM model, introducing CBAM modules into both the generator and discriminator to achieve synergistic optimization of channel and spatial attention. **Figures 14**–**17** showcase the generated results of tea and coffee disease images.

In the generation of tea white star disease, SinGAN-CBAM exhibits more evenly distributed spots, clearer edges, and higher morphological fidelity. In the generation of algal spot disease, the position and shape of the spots are closer to the real images, with the overall texture of the leaf being well-preserved without noticeable distortion. For coffee leaf rust, SinGAN-CBAM effectively reduces the adhesion between spots and enhances the naturalness of edge textures. In the generation of leaf miner damage, the edges of the patches appear naturally rough with distinct color layers, clear traces of insect damage, and a more naturally blended background.

To verify the effectiveness of CBAM, this paper quantitatively evaluates SinGAN and SinGAN-CBAM using the same metrics, with the results shown in **Tables 7** and **8**.

**Table 7:** Quantitative evaluation of the quality of tea generation images.

Evaluation index	SinGAN	SinGAN-CBAM
SSIM	0.682	0.826
MSE	46.97	26.88
PSNR	31.41	33.84
Tenengrad	0.567	0.829

**Table 8:** Quantitative evaluation of the quality of coffee generation images.

Evaluation index	SinGAN	SinGAN+ CBAM
SSIM	0.636	0.675
MSE	48.48	37.46
PSNR	31.27	32.39
Tenengrad	0.454	0.782

In tea leaf images, the incorporation of CBAM leads to a significant improvement in SSIM, with MSE decreasing by 42.8%, an increase in PSNR, and a substantial rise in the Tenengrad value, indicating notable advancements in structure, noise control, and edge clarity of the generated images. In coffee leaf images, MSE is reduced by 22.7%, with improvements in both PSNR and Tenengrad, suggesting an overall enhancement in generation quality. The results demonstrate that the dual-dimensional attention mechanism of CBAM effectively boosts the model’s ability to perceive diseased areas and improves detail reproduction accuracy.

### 3.5 Comparative analysis of two attention mechanisms

**Figure 18:**
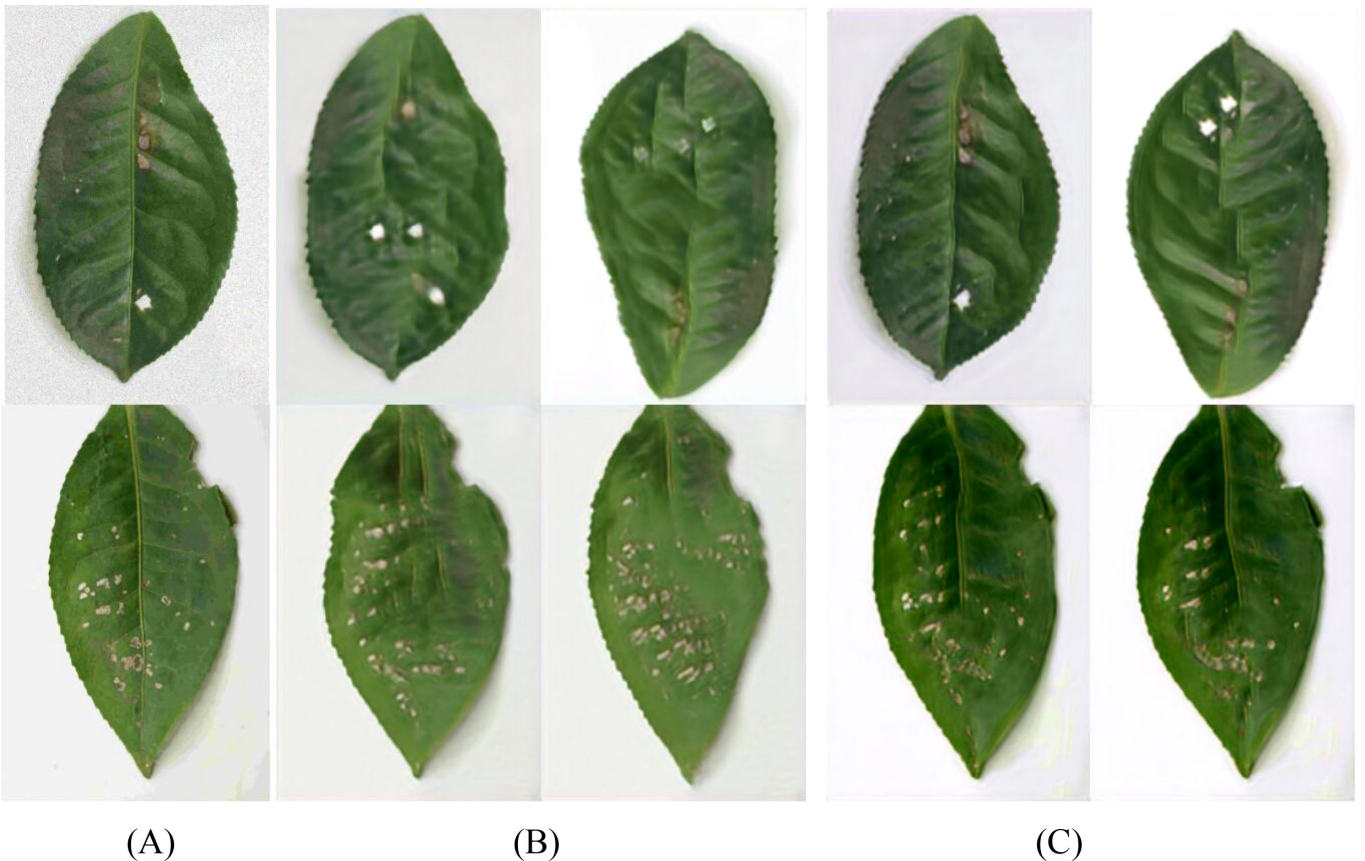
Tea scab generated image. **(A) **Original image; **(B) **SinGAN generated images; **(C)** SinGAN-CBAM generated images.

**Figure 19:**
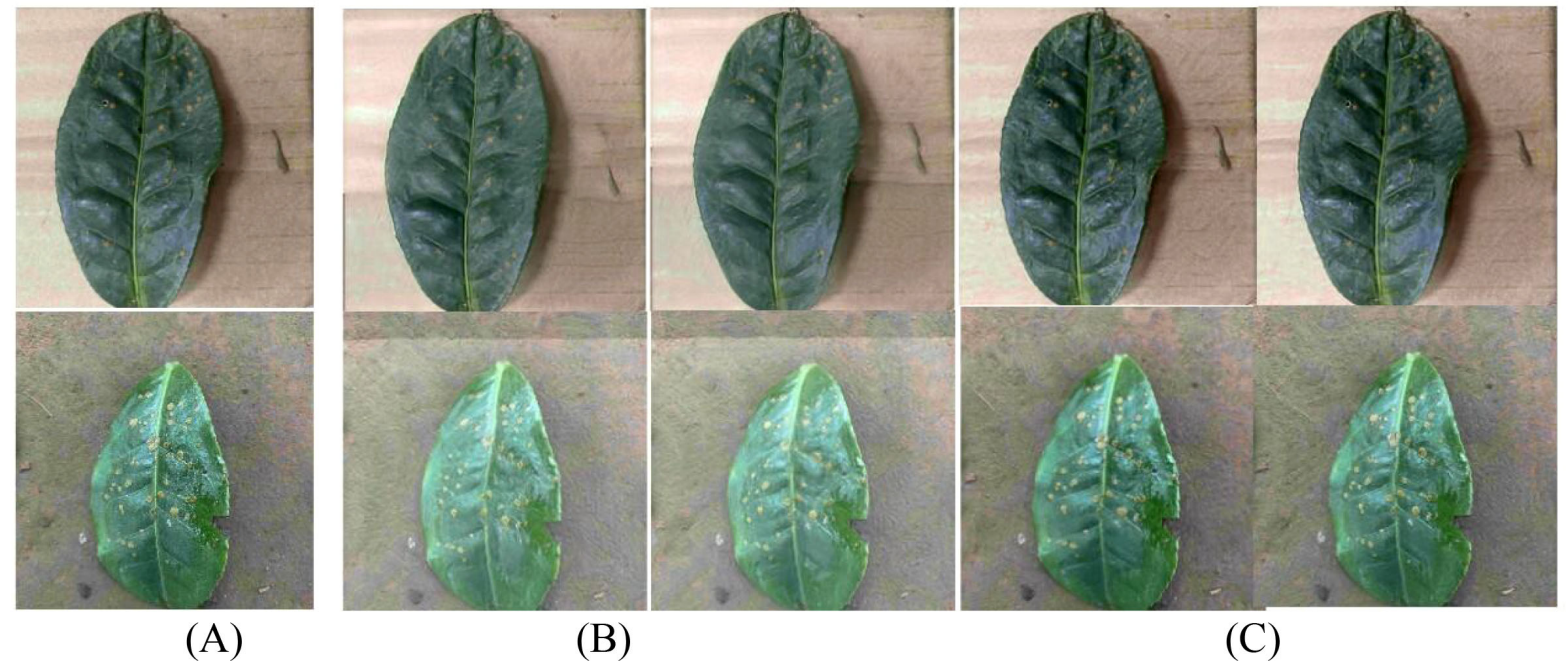
The image of algae spot disease in tea leaves. **(A)** Original image; **(B)** SinGAN generated images; **(C)** SinGAN-CBAM generated images.

**Figure 20:**
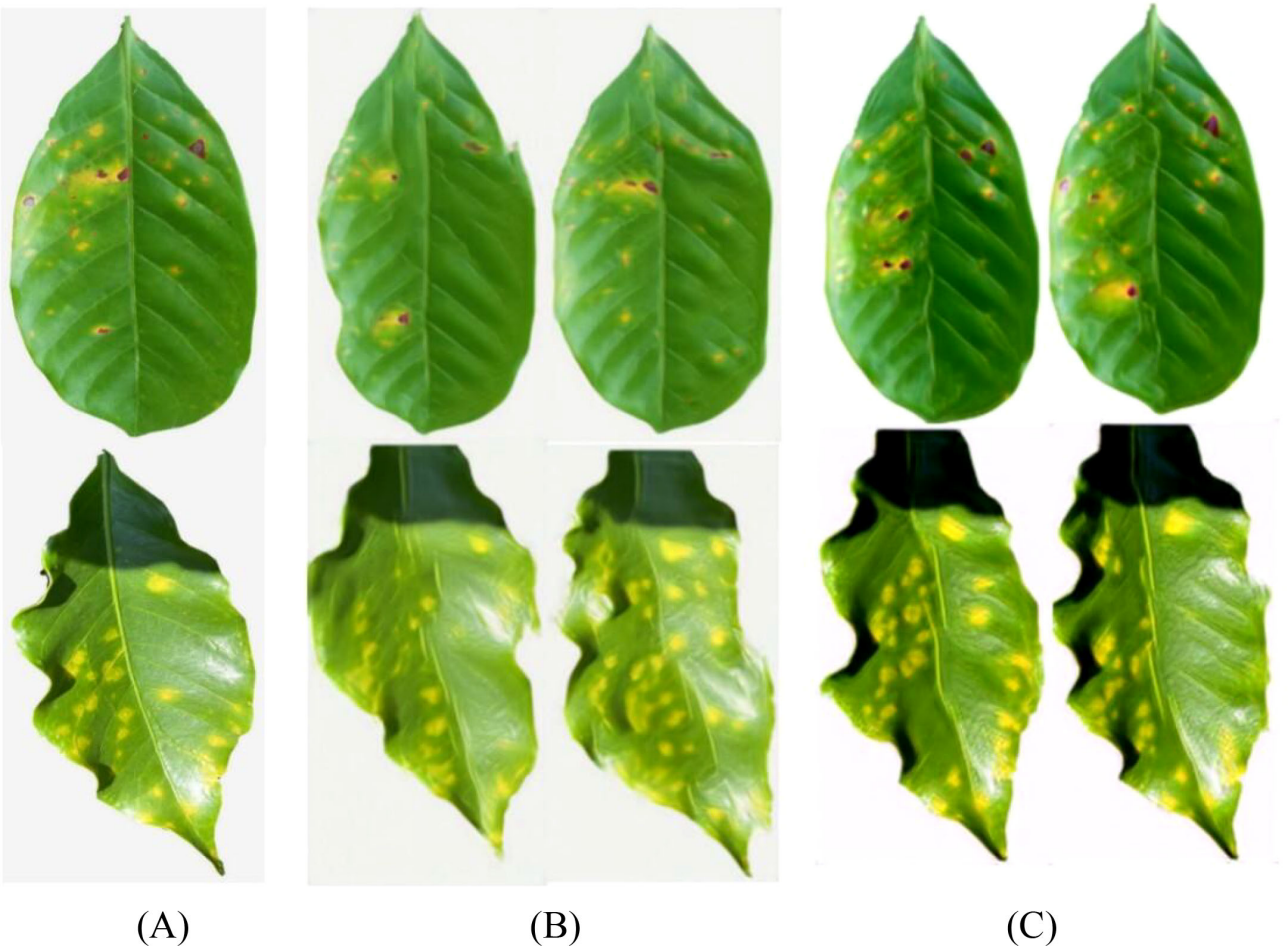
Coffee leaf rust image. **(A)** Original image; **(B)** SinGAN generated images; **(C)** SinGAN-CBAM generated images.

**Figure 21:**
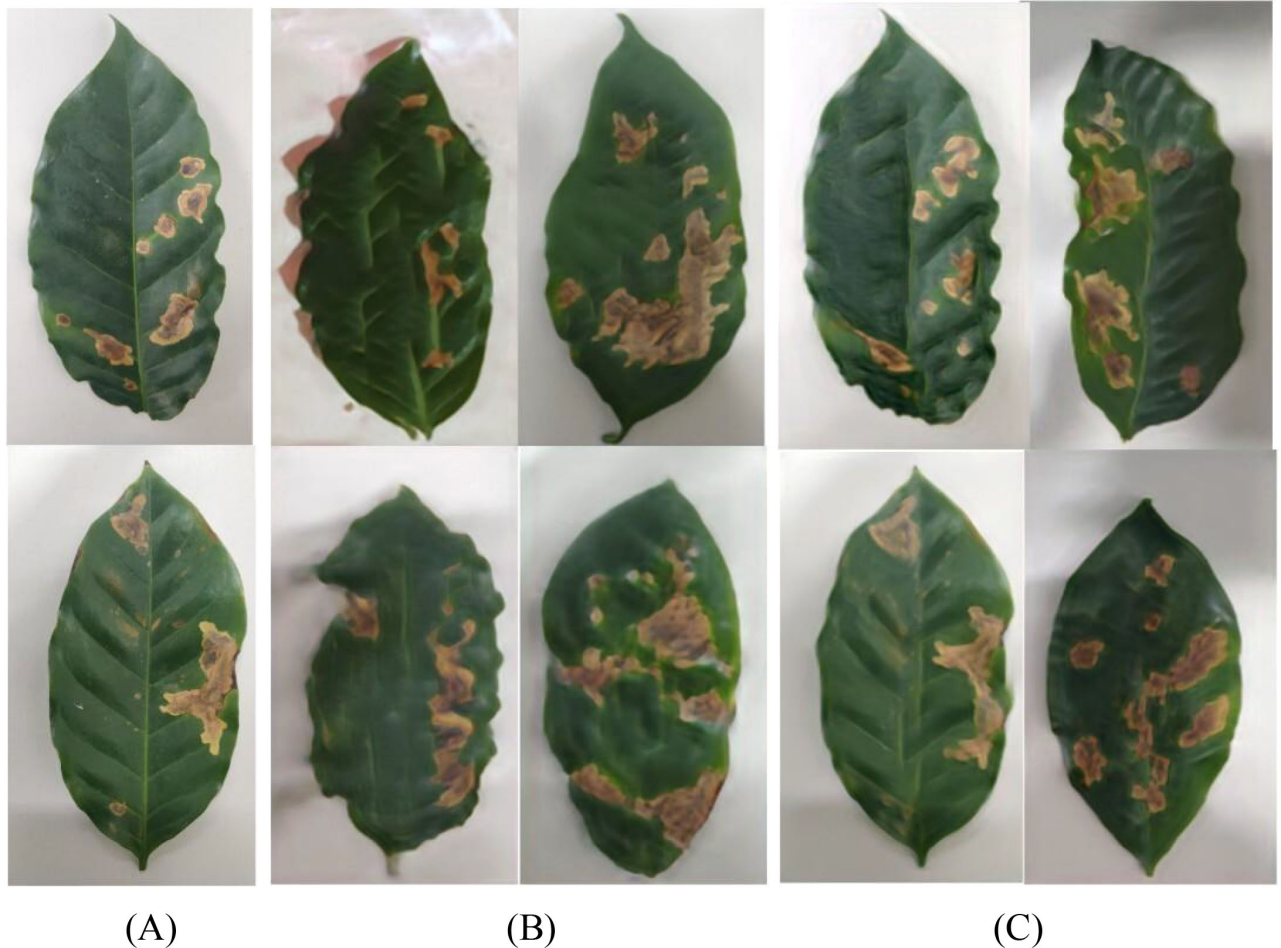
Coffee leaf miner moth generation image. **(A)** Original image; **(B)** SinGAN generated images; **(C)** SinGAN-CBAM generated images.

To intuitively compare the improvement effects of SELayer and CBAM, this article selects typical disease images for comparative analysis, with the results shown in **Figures 18**–**21**. From a visual perspective, SinGAN-CBAM outperforms SinGAN-SE in terms of spot edge clarity, distribution naturalness, and texture coherence, particularly excelling in the restoration of spatial structures.

Further aggregating the quantitative metrics of the two improved models, the results are shown in **Table 9**. In tea leaf image generation, SinGAN-CBAM shows improvements over SinGAN-SE with an increase of 15.7% in SSIM, a decrease of 18.1% in MSE, an increase of 2.6% in PSNR, and an increase of 24.8% in Tenengrad, with all metrics being superior. In coffee images, although SinGAN-SE has a slightly higher SSIM (possibly due to its ability to enhance low-resolution textures), SinGAN-CBAM performs better in terms of MSE (with a decrease of 9.0%), PSNR (an increase of 1.3%), and Tenengrad (an increase of 30.0%).

**Table 9:** Evaluation indicators for the quality of tea and coffee images generated by two models.

Study subjects	Index	SinGAN-SELayer	SinGAN-CBAM
Tea	SSIM	0.714	0.826
	MSE	32.83	26.88
	PSNR	32.97	33.84
	Tenengrad	0.664	0.829
Coffee	SSIM	0.691	0.675
	MSE	41.18	37.46
	PSNR	31.98	32.39
	Tenengrad	0.602	0.782

Overall, SinGAN-CBAM demonstrates a clear advantage in the overall quality of generated tea and coffee disease images, which fully validates the effectiveness of the dual-dimension attention mechanism in enhancing the realism and detail fidelity of generated images.

### 3.6 Verification of downstream classification performance of generated images

To evaluate the usability and semantic fidelity of generated images in practical agricultural disease recognition tasks, this paper further conducts disease classification experiments based on the YOLOv8 model. The experiment selects images generated by SinGAN, SinGAN-SE, and SinGAN-CBAM models as input data, indirectly reflecting the quality of the generated images through classification performance. Although SinGAN is inherently a single-image generative model, we apply it in a ‘few-shot augmentation’ pipeline: for each of the N real images in a disease class, an independent SinGAN instance is trained. The union of all synthetic outputs constitutes the augmented dataset.

During the image generation phase, 10 new samples are generated for each original training image. For the tea dataset, which includes four disease categories, each category originally has 100 samples, which are expanded to 1000 images after generation. The coffee dataset contains three disease categories, with each category similarly generating 1000 synthetic images from 100 original images. All generated images are divided into training, validation, and test sets at an 8:1:1 ratio for the training and evaluation of the YOLOv8 model. Classification performance is evaluated using precision, recall, and F1-score as metrics, with results shown in **Table 10**.

**Table 10:** Each model generates image disease classification results.

Tea and coffee diseases	Model	F1-score	Recall	Precision
Tea leaf white spot disease	SinGAN	0.80	0.85	0.76
	SinGAN-SELayer	**0.86**	**0.91**	**0.81**
	SinGAN-CBAM	**0.95**	**0.94**	**0.96**
Tea Anthracnose	SinGAN	0.79	0.83	0.75
	SinGAN-SELayer	**0.86**	**0.90**	**0.82**
	SinGAN-CBAM	**0.93**	**0.92**	**0.94**
Tea algae spot	SinGAN	0.73	0.78	0.69
	SinGAN-SELayer	**0.81**	**0.86**	**0.77**
	SinGAN-CBAM	**0.93**	**0.93**	**0.94**
Tea leaf blight	SinGAN	0.79	0.83	0.76
	SinGAN-SELayer	**0.86**	**0.88**	**0.84**
	SinGAN-CBAM	**0.93**	**0.92**	**0.94**
Coffee rust	SinGAN	0.79	0.80	0.79
	SinGAN-SELayer	**0.88**	**0.87**	**0.89**
	SinGAN-CBAM	**0.91**	**0.90**	**0.93**
Coffee leaf spot	SinGAN	0.82	0.85	0.80
	SinGAN-SELayer	**0.88**	**0.92**	**0.85**
	SinGAN-CBAM	**0.94**	**0.93**	**0.96**
Coffee leaf miner	SinGAN	0.88	0.87	0.90
	SinGAN-SELayer	**0.94**	**0.98**	**0.91**
	SinGAN-CBAM	**0.98**	**0.99**	**0.98**

The bolded figures in the table are to better highlight the outstanding results (values) of SinGAN-SELayer and SinGAN-CBAM.

The experimental results indicate that images synthesized by generative models incorporating attention mechanisms exhibit superior recognition performance in downstream classification tasks. Compared to the original SinGAN, both SinGAN-SE and SinGAN-CBAM showed improved classification metrics, with SinGAN-CBAM demonstrating the best overall performance.

Specifically, across all disease types, SinGAN-CBAM achieved the highest levels of precision, recall, and F1-score. For instance, regarding the coffee leaf miner disease, its precision improved from 0.90 with the original SinGAN to 0.98, with a recall nearing 1.00 and an F1-score reaching 0.98. This indicates that the images generated by this model not only retain critical lesion features but also possess a high degree of semantic consistency, making them accurately identifiable by recognition models. In the case of tea blight, SinGAN-CBAM achieved an precision rate of 0.94, suggesting that the lesions it generates are clear in shape and have distinctive features that are easily recognizable by classifiers.

The improvement effects of the attention mechanism are particularly evident in categories that are challenging to distinguish. Images of tea algae spot disease generated by the original SinGAN had a classification precision of merely 0.69 and an F1-score of 0.73, indicating blurred lesion boundaries and low differentiation from healthy tissue in the generated images. Post-introduction of attention mechanisms, classification performance significantly improved: SinGAN-SE increased the F1-score for this category to 0.81, while SinGAN-CBAM further raised it to 0.93, demonstrating its effective enhancement of modeling capabilities for subtle lesion areas through dual-channel and spatial attention mechanisms, thereby improving the discriminability of disease features.

Similarly, on diseases such as anthracnose in tea characterized by indistinct edges and complex color gradients, SinGAN-CBAM exhibited stronger feature restoration abilities. Although SinGAN-SE enhanced key feature responses through channel attention, it was somewhat lacking in spatial positioning and local detail depiction, resulting in slightly lower classification performance than SinGAN-CBAM.

Comprehensive analysis shows that the CBAM module, by collaboratively optimizing channel importance and spatial attention regions, ensures that generated images surpass other models in terms of texture clarity, natural distribution of lesions, and structural consistency. Consequently, this significantly enhances the recognition accuracy and robustness of downstream classification models. These results further validate that high-quality generated images should not only possess visual authenticity but also retain semantic features useful for identification. In this regard, SinGAN-CBAM demonstrates significant advantages.

## 4 Discussion

Addressing the scarcity of disease image data for tea and coffee leaves, this study proposes an image generation framework that integrates multi-scale generation with attention mechanisms. Experimental results demonstrate that under small sample conditions, SinGAN exhibits a clear advantage in generation quality over traditional GANs and Fast-GAN, capable of learning texture and structural priors from a single image to generate visually natural and semantically consistent disease samples. This characteristic makes it highly applicable for low-resource and hard-to-collect image enhancement tasks in agriculture, contrasting sharply with existing research emphasizing large-scale data-driven image generation methods (Tan et al., 2023).

Further, by introducing attention mechanisms to improve SinGAN, this paper explores the potential of channel attention (SELayer) and dual-dimension attention (CBAM) in enhancing the expression ability of disease features. The results show that SELayer can enhance the model’s response intensity to lesion area features by adaptively adjusting channel weights, improving spot boundary clarity and color contrast, which aligns with Hu et al.’s (2018) observation of channel feature recalibration effects in image classification tasks. However, its effect is limited to the channel dimension, making it difficult to effectively model the spatial distribution patterns of lesions, thus limiting further improvement in complex texture restoration.

In contrast, CBAM achieves collaborative focusing on “important features” and “key locations” during generation by jointly optimizing channel and spatial attention. Experiments indicate that SinGAN-CBAM more closely mimics real disease morphology in terms of rust spot distribution and leaf miner trail orientation, significantly enhancing the semantic fidelity of images. These findings echo those of Woo et al. (2018), who found that CBAM improves localization accuracy in object detection, further validating the effectiveness of spatial attention mechanisms in fine-grained visual tasks.

Although SinGAN-CBAM demonstrates excellent performance on the tea leaf disease dataset, its generation quality on coffee disease images is notably limited, primarily due to the high heterogeneity in image acquisition conditions within the original dataset. Specifically, among the 400 original coffee disease images used in this study, approximately 72.6% (290 images) have resolutions lower than 1920×1080 pixels, and nearly 79% (316 images) exhibit non-standard aspect ratios (i.e., neither 16:9 nor 9:16), with some images even suffering from severe stretching or compression. This inconsistency poses a significant challenge for SinGAN-based models, which rely on internal statistical priors learned from a single image for multi-scale reconstruction. Low input resolution restricts the model’s ability to capture high-frequency textures, while unnatural aspect ratios induce spatial structural distortions during generation, ultimately compromising the fidelity of lesion morphology. This issue highlights a key limitation of current single-image generative models when applied to non-standardized agricultural imagery and aligns with recent findings that “generative models are highly sensitive to input geometric properties” (Wang and Ponce, 2021). To mitigate this problem, we recommend incorporating a standardized preprocessing pipeline in practical applications: first applying center cropping or lesion-aware intelligent cropping, followed by resizing all images to a fixed resolution. This strategy preserves critical disease features while enhancing structural consistency in the input data. In contrast, transfer learning or cross-domain generative approaches have demonstrated greater robustness in handling heterogeneous data (Wang et al., 2020), suggesting that future work should explore integrating pretraining strategies with multi-scale generation frameworks to improve model generalization and real-world applicability.

In conclusion, by embedding attention mechanisms into a multi-scale generation framework, this study achieves higher-quality disease image synthesis under small sample conditions. Experimental results not only verify the feasibility of SinGAN in agricultural image generation but also reveal the potential of attention mechanisms in enhancing semantic consistency. However, the model still has limitations regarding cross-resolution adaptability, and future work could explore integrating transfer learning, self-supervised priors, or conditional generation strategies to further enhance the model’s practicality and generalization capabilities.

## 5 Conclusion

Addressing the challenges of scarce data, high acquisition costs, and insufficient sample diversity in tea and coffee leaf disease image datasets, this study constructs and validates an image generation framework based on the integration of multi-scale generation and attention mechanisms. By comparing the performance of three generative models—GAN, Fast-GAN, and SinGAN—under few-shot conditions, the results demonstrate that SinGAN significantly outperforms traditional generative models in generation quality and structural fidelity due to its ability to learn texture and structural priors from a single image, making it more suitable for low-resource data augmentation tasks in agriculture.

The proposed SinGAN-CBAM framework exhibits strong performance and practical potential in both few-shot agricultural image generation and downstream classification tasks. It achieves significantly better results than SinGAN and SinGAN-SE across multiple metrics: in image quality assessment (e.g., SSIM, PSNR, and Tenengrad) and in classification performance (e.g., precision, recall, and F1-score). Notably, it excels in recognizing complex textures (e.g., algal spot disease) and subtle lesions (e.g., leaf miner damage), demonstrating that the generated samples possess high semantic fidelity and usability. This framework thus offers an efficient and cost-effective data augmentation solution for plant disease identification. Future work may explore incorporating transfer learning, cross-scale alignment mechanisms, or conditional generation strategies to further enhance the model’s generalization capability and robustness in real-world deployment scenarios.

## Data Availability

The raw data supporting the conclusions of this article will be made available by the authors, without undue reservation.
